# Predictive Description of Condensed-Phase Hyperfine Coupling Constants Relies on Dielectric Screening of Long-Range Exchange

**DOI:** 10.1021/acs.jpclett.6c02938

**Published:** 2026-10-14

**Authors:** Slater T. Bakenhaster, Barry D. Dunietz

**Affiliations:** Department of Chemistry and Biochemistry, 4229 Kent State University, Kent, Ohio 44242, United States

## Abstract

Hyperfine coupling constants (HFCCs) provide a direct probe of the interaction between electron and nuclear spins and are important descriptors in the design of molecular spin qubits for quantum information science. Accurate prediction of HFCCs in condensed-phase environments requires describing the influence of environmental polarization on the underlying spin density. Here, we demonstrate that dielectric screening of the exchange interaction in the long-range limit of range-separated hybrid (RSH) functionals, as introduced in SRSH, substantially improves calculated ^1^H and ^13^C HFCCs. Systematic analysis of arene radical anions examines the effects of dielectric screening, basis-set choice, and the weight of short-range exact exchange, demonstrating the improved accuracy achieved by using SRSH–PCM for calculating HFCCs. The applicability of SRSH–PCM is further demonstrated for semiquinone derivatives, where the screened treatment captures changes in the HFCCs across different dielectric environments. These results establish dielectric screening of long-range exchange as key for accurately predicting condensed-phase hyperfine interactions and their response to changes in the molecular environment, providing a basis for reliably extending such calculations to more complex EPR-active systems.

## Graphical abstract

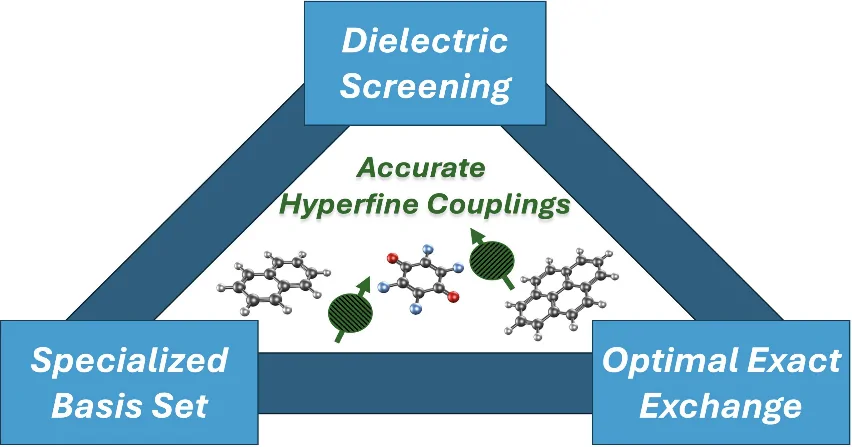

Electron paramagnetic resonance (EPR) spectroscopy is an important tool across a broad range of chemical applications.^1–3^ By probing the interactions of unpaired electron spins with their molecular environment, EPR provides detailed information about the electronic structure of open-shell systems. In particular, EPR has become increasingly important in quantum information science, where the design of molecular electron-spin qubits requires an understanding of the interactions that govern spin-state properties and coherence.^4–7^ Among the central quantities measured by EPR are hyperfine coupling constants (HFCCs), which characterize the interaction between an unpaired electron spin and the nuclear spins of the molecule. HFCCs can strongly influence the properties and performance of molecular spin-qubit candidates.^8,9^ They are also important in studies of redox processes, electron transfer, and photosynthetic systems.^10–13^ Accurate and computationally accessible prediction of HFCCs is therefore important for interpreting EPR spectra and relating molecular electronic structure to experimentally observed spin properties.^14,15^

Density functional theory (DFT) has been extensively applied to the calculation of HFCCs. O’Malley and collaborators^16–19^ used DFT to investigate HFCCs in quinone radicals, including the effects of electron transfer and hydrogen bonding. More recently, experimental and computational studies of HFCCs in flavin semiquinone radicals have demonstrated their ability to probe the underlying spin-density distribution.^12^ Considerable effort has also been devoted to developing basis sets specifically suited for the accurate description of HFCCs.^20,21^ DFT calculations of HFCCs have also been extended to molecular spin-qubit candidates. Cardona et al.,^22^ for example, examined the performance of different density functionals and relativistic treatments for transition-metal molecular qubits.

An additional challenge for condensed-phase systems is the polarization of the surrounding dielectric environment.^23^ Isotropic HFCCs depend directly on the spin density at the nucleus, making their calculated values sensitive to environment-induced changes in electronic structure.^21,24^ Continuum solvation models account for the electrostatic polarization of the environment but do not by themselves incorporate dielectric screening into the exchange interaction.^25,26^ Screened range-separated hybrid (SRSH) functionals provide a physically motivated framework for dielectric screening of the exchange interaction in the long-range limit.^27–30^ This raises the question of whether dielectric screening of long-range exchange can systematically improve the description of HFCCs in solution and how this effect depends on other important components of the calculation, particularly the weight of short-range exact exchange and the choice of basis set.^21,31^

Here, we examine whether dielectric screening of the exchange interaction in the long-range limit of RSH functionals improves the prediction of condensed-phase HFCCs. We first use three arene radical anions to assess ^13^C and ^1^H HFCCs and to establish the effects of dielectric screening, the weight of short-range exact exchange, and basis-set choice. We then examine the applicability of the resulting SRSH–PCM treatment to *p*-benzoquinone derivatives and, importantly, test whether it captures the response of their HFCCs to changes in the dielectric environment.

The hyperfine interaction between an electron spin and a nuclear spin is described by the spin Hamiltonian

1 $$H_{\mathrm{HFCC}}=S\cdot A\cdot I$$

where **S** and **I** are the electron and nuclear spin operators, respectively, and **A** is the hyperfine coupling tensor.^32–34^ The hyperfine tensor can be decomposed into isotropic and anisotropic components:

2 $$A=A_{iso}1+T$$

where **1** is the identity matrix and **T** is the traceless anisotropic component.^3,35^ The isotropic hyperfine coupling, *A*_iso_, is given by one-third of the trace of **A**:^3,36^

3 $$A_{iso}=\frac{A_{11}+A_{22}+A_{33}}{3}$$

In the nonrelativistic approximation used here, *A*_iso_ is determined by the Fermi contact interaction:

4 $$A_{iso}=A_{FC}\left(N\right)=\frac{4\pi }{3\left\langle S_{z}\right\rangle }g_{e}g_{N}\beta_{e}\beta_{N}\rho \left(R_{N}\right)$$

where *g*_e_ and *g*_N_ are the electron and nuclear *g* factors, β_e_ and β_N_ are the corresponding magnetons, and ρ­(**R**_*N*_) is the spin density at nucleus *N*.^1,22,37^ Consequently, accurate prediction of *A*_iso_, and therefore of HFCCs, remains challenging because of its sensitivity to the calculated spin density at the nucleus, motivating continued development and assessment of electronic-structure approaches for these properties.^37–39^

RSH functionals partition the Coulomb interaction into components treated with exact and semilocal exchange, with the relative fractions varying between the short- and long-range (SR and LR) limits:

5 $$\frac{1}{r}=\frac{\alpha +\beta \;erf\left(\omega r\right)}{r}+\frac{1-\left[\alpha +\beta \;erf\left(\omega r\right)\right]}{r}$$

where α and α + β define the fractions of exact exchange in the SR and LR limits, respectively, and ω is the range-separation parameter that determines the switching between these limits.^40–42^ In the generalized Kohn–Sham (GKS) framework^43,44^ the exchange–correlation energy of the RSH functional can be expressed as^42,45,46^

6 $$E_{\mathrm{XC}}^{RSH}=\alpha E_{X,HF}^{SR}+\left(1-\alpha \right)E_{X,PBE}^{SR}+\left(\alpha +\beta \right)E_{X,HF}^{LR},+\left(1-\alpha -\beta \right)E_{X,PBE}^{LR}+E_{C,PBE}$$

For a specified α, the range-separation parameter ω is determined by optimal tuning using the generalized Koopmans condition:^27,47,48^

7 $$J\left(\omega \right)={\left[\varepsilon_{HOMO}\left(\omega \right)+IP\left(\omega \right)\right]}^{2}+{\left[\varepsilon_{LUMO}\left(\omega \right)+EA\left(\omega \right)\right]}^{2}$$

For systems with a negative calculated EA, tuning was based on the IP–HOMO condition alone.^27^ In RSH–PCM, solvent polarization is introduced through the PCM reaction field, while the LR exact-exchange contribution remains unscreened.^28,49–51^

For condensed-phase systems, the SRSH framework incorporates dielectric screening directly into the LR electron–electron interaction by setting

8 $$\alpha +\beta =\frac{1}{\varepsilon }$$

where ε is the dielectric constant of the surrounding medium.^27,52^ The corresponding GKS exchange–correlation energy becomes^53^

9 $$E_{\mathrm{XC}}^{SRSH}=\alpha E_{X,HF}^{SR}+\left(1-\alpha \right)E_{X,PBE}^{SR}+\frac{1}{\varepsilon }E_{X,HF}^{LR}+\left(1-\frac{1}{\varepsilon }\right)E_{X,PBE}^{LR}+E_{C,PBE}$$

Accordingly, dielectric screening sets the LR exact-exchange weight to 1/ε, with a complementary LR semilocal-exchange weight of 1 – 1/ε. Combined with PCM, SRSH–PCM accounts for the dielectric environment through both the continuum reaction field and screening of the LR exchange interaction.^27,51,54–57^

We evaluate SRSH–PCM for ^13^C and ^1^H HFCCs of the naphthalene, anthracene, and pyrene radical anions, with comparison to experiment and the fixed-parameter ωB97X-D functional. RSH–PCM calculations are included to highlight the effect of dielectric screening of the exchange interaction in the LR limit. To account for the sensitivity of HFCCs to the exact-exchange weight,^31,58,59^ we varied the SR exact-exchange weight α from 0.00 to 0.50 in increments of 0.05. For each value of α, ω was optimally tuned in the gas phase and subsequently used without further tuning in both RSH–PCM and SRSH–PCM calculations.

The basis-set dependence of the calculated HFCCs was examined using the general-purpose 6-31+G­(d)^60^ and cc-pVDZ^61^ basis sets together with three basis sets specifically designed for magnetic properties: pcH-1, 6-31+G­(d)-J, and ccJ-pVDZ.^21^ Accurate calculation of the Fermi-contact contribution requires sufficient flexibility in the core region, motivating the use of these specialized basis sets. The pcH-1 basis set is based on the polarization-consistent basis-set framework,^21,62^ and the 6-31+G­(d)-J and ccJ-pVDZ basis sets are derived from the Pople and Dunning basis-set families, respectively. These basis sets were originally developed for the calculation of indirect nuclear spin–spin coupling constants,^20,63^ for which an accurate description of the electronic structure near the nucleus is likewise important.

Geometry optimizations for all systems were performed at the ωB97X-D/cc-pVDZ level of theory.^61,64^ Each system was optimized in its corresponding charge state using PCM with the relevant dielectric constant, ε. For selected systems, additional calculations including explicit solvent molecules (denoted by ^a^) were performed in addition to the PCM-only calculations. The solvent molecules were initially positioned to form hydrogen-bonding interactions with the solute, followed by optimization of the complete system at the same level of theory. The coordinates of the optimized geometries are provided in Supporting Information (SI) section 3. All calculations were performed using Q-Chem 6,^65^ with sample inputs given in SI section 3. Specialized basis sets were obtained from the Basis Set Exchange.^66–68^

We first compare the calculated ^13^C and ^1^H HFCCs with experimental EPR measurements for the naphthalene, anthracene, and pyrene radical anions.^36,69–72^ The molecular structures and atom numbering are shown in Figure S1. For consistency across the arene benchmark, each anionic arene was modeled in dimethoxyethane (DME) (ε = 7.2).^73^ The experimental *A*_iso_ values considered in this work range from 0.010 to 0.876 mT. For each arene and nucleus type, the mean absolute error (MAE) was calculated by averaging the absolute errors over the symmetry-distinct HFCCs. Accordingly, symmetry-equivalent nuclei contribute a single HFCC to the MAE. For example, although naphthalene contains ten carbon atoms, it has only three symmetry-distinct ^13^C HFCCs. The individual calculated and experimental *A*_iso_ values used in the analysis are provided in SI Section 2.^69,70,74–76^ For each semiquinone, the ^13^C HFCC MAE was similarly calculated by averaging the absolute errors for the two symmetry-distinct carbon sites: one corresponding to the four equivalent noncarbonyl carbon atoms and the other to the two equivalent carbonyl carbon atoms.^77^

Figure 1 compares SRSH–PCM with RSH–PCM at α = 0.20 and 0.35 using the cc-pVDZ (Figure 1a–c) and pcH-1 (Figure 1d–f) basis sets. In all cases, SRSH–PCM yields lower MAEs for both ^13^C and ^1^H HFCCs than RSH–PCM. Reducing the SR exact-exchange weight from α = 0.35 to 0.20 further lowers the MAEs, while the comparison between cc-pVDZ and pcH-1 reveals a pronounced basis-set dependence, particularly for ^13^C. These results demonstrate the sensitivity of the calculated HFCCs to dielectric screening, SR exact exchange, and basis-set choice, which we examine systematically next.

**Figure 1:**
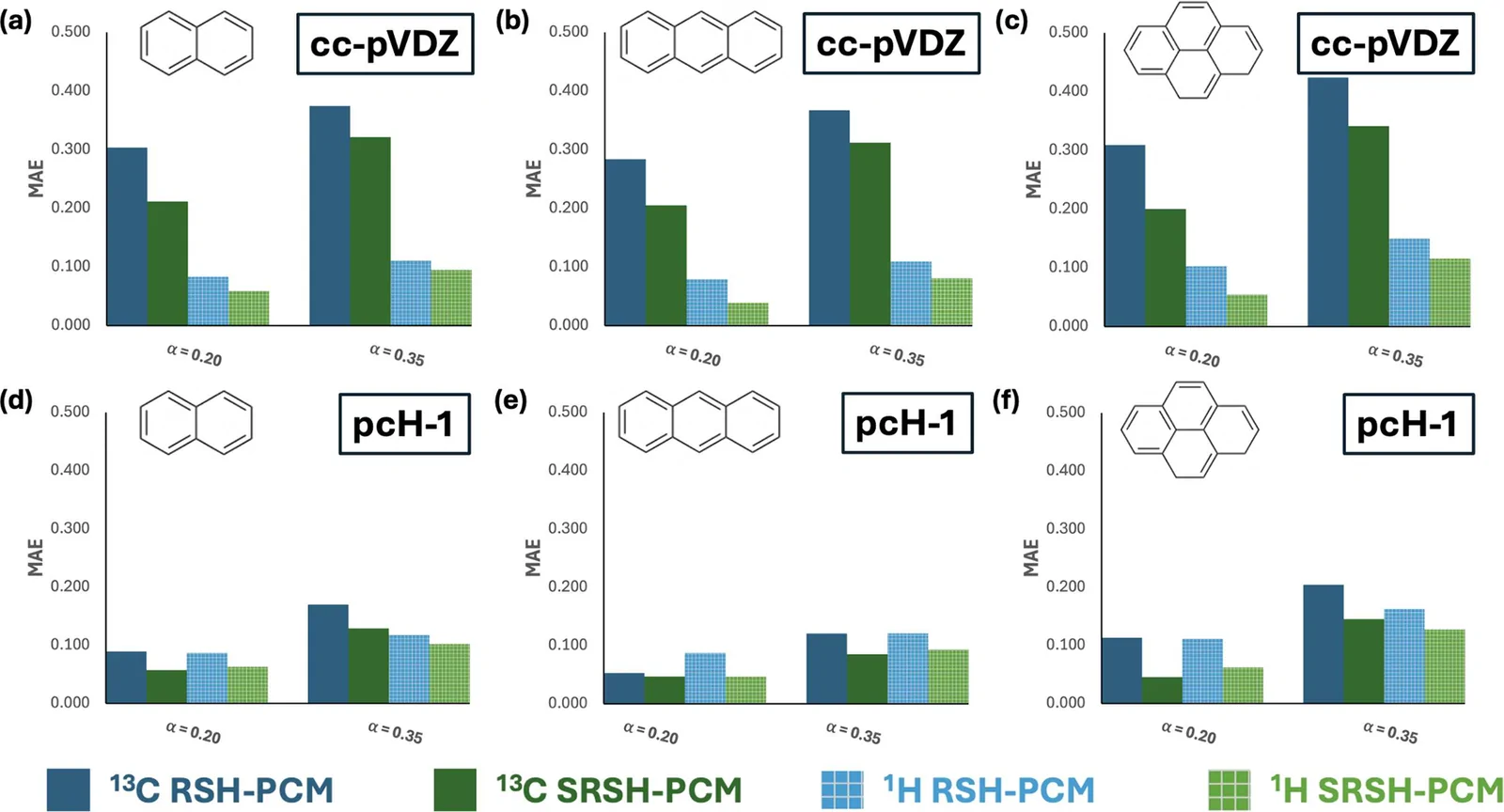
MAEs of the ^1^H and ^13^C isotropic hyperfine coupling constants (*A*_iso_), averaged over the corresponding atomic sites, for (a, d) naphthalene, (b, e) anthracene, and (c, f) pyrene, calculated using RSH–PCM and SRSH–PCM with α = 0.20 and 0.35 and the (a–c) cc-pVDZ and (d–f) pcH-1 basis sets. Representative calculated HFCCs and the corresponding experimental values^36,69–72^ are listed in Tables S10 and S14.

The dependence of HFCC accuracy on the SR exact-exchange weight was examined over the range α = 0.00–0.50 for both RSH–PCM and SRSH–PCM. Figure 2 shows representative α scans for cc-pVDZ, 6-31+G­(d)-J, and pcH-1. Results for all five basis sets, together with the gas-phase-tuned ω values at each α, are provided in SI section 1 (Tables S1–S5).

**Figure 2:**
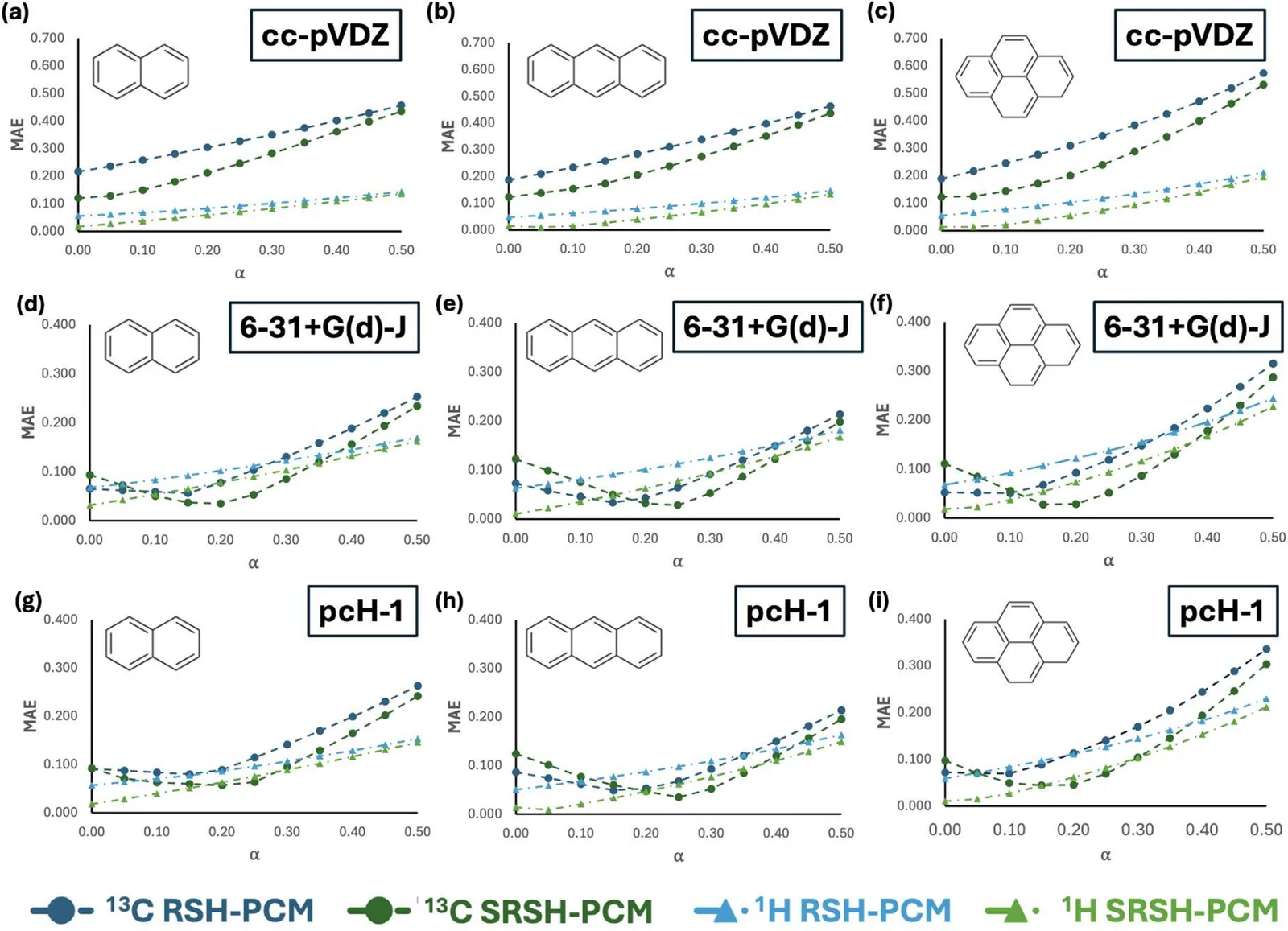
MAEs of ^1^H and ^13^C isotropic hyperfine coupling constants (*A*_iso_), averaged over the corresponding atomic sites, as a function of α for (a, d, g) naphthalene, (b, e, h) anthracene, and (c, f, i) pyrene, calculated using RSH–PCM and SRSH–PCM with the (a–c) cc-pVDZ, (d–f) 6-31+G­(d)-J, and (g–i) pcH-1 basis sets. Representative calculated HFCCs and the corresponding experimental values^36,69–72^ are listed in Tables S10, S12, and S14.

For the general-purpose cc-pVDZ and 6-31+G­(d) basis sets, the lowest MAEs for both ^1^H and ^13^C HFCCs are found at α = 0, with the errors increasing as the SR exact-exchange weight increases. SRSH–PCM nevertheless yields lower MAEs than RSH–PCM across the α scan. For ^1^H, the lowest SRSH–PCM MAEs with cc-pVDZ range from 0.0137 to 0.0175 mT, approximately three to four times smaller than the corresponding RSH–PCM values. In contrast, the ^13^C MAEs remain on the order of 0.1 mT, comparable in magnitude to the calculated HFCCs. The same qualitative behavior is obtained with 6-31+G­(d) (Table S2), indicating that neither of the considered general-purpose basis sets provides an adequate description of the ^13^C HFCCs.

The considerable ^13^C errors obtained with the general-purpose basis sets motivate the use of HFCC-specialized basis sets. Figure 2d–f and Figure 2g–i show the α dependence for 6-31+G­(d)-J and pcH-1, respectively. For ^1^H HFCCs, the specialized basis sets produce only modest changes relative to the general-purpose basis sets, while SRSH–PCM continues to yield lower MAEs than RSH–PCM. For example, for the anthracene anion at α = 0, the SRSH–PCM MAEs vary only from 0.010 to 0.024 mT across the five basis sets.

In contrast to ^1^H HFCCs, the accuracy of ^13^C HFCCs is strongly influenced by the choice of basis set. For the HFCC-specialized basis sets, Table 1 and Figure 2 show that the smallest SRSH–PCM MAEs are consistently lower than the corresponding RSH–PCM minima for all three arenes. The α dependence shows a semiparabolic behavior for both methods, with the RSH–PCM MAE minima shifted toward lower α values relative to SRSH–PCM. Consequently, RSH–PCM can yield slightly lower MAEs at very small α, whereas SRSH–PCM is generally more accurate beyond this crossover and throughout the commonly employed α = 0.20–0.45 range. The smallest SRSH–PCM MAEs are typically found with α = 0.20–0.25.

**Table 1:** MAEs of the Calculated ^13^C HFCCs, Averaged over All Carbon Sites for Each Arene, Obtained with RSH–PCM, SRSH–PCM, and *ω*B97X-D Using Different Basis Sets; Representative Calculated HFCCs and the Corresponding Experimental Values^36,69–72^ Are Listed in Tables S12–S14

	naphthalene			anthracene			pyrene		
method	6-31+G(d)-J	ccJ-pVDZ	pcH-1	6-31+G(d)-J	ccJ-pVDZ	pcH-1	6-31+G(d)-J	ccJ-pVDZ	pcH-1
ωB97X-D	0.10955	0.11777	0.09984	0.09251	0.09376	0.06996	0.09728	0.10369	0.09164
RSH-PCM	0.05600^b^	0.08507^c^	0.07994^b^	0.03358^b^	0.05119^c^	0.04903^b^	0.05074^a^	0.07443^a^	0.06977^a^
SRSH-PCM	0.03491^c^	0.06157^d^	0.05723^c^	0.02825^c^	0.04010^d^	0.03487^c^	0.02786^b^	0.04851^b^	0.04468^b^

a α = 0.10,

b α = 0.15.

c α = 0.20.

d α = 0.25.

The calculations were repeated using the larger 6-311+G­(d)-J and pcH-2 basis sets; Tables S6 and S7 provide the corresponding MAEs and ω values. Increasing the basis-set size from double-ζ to triple-ζ produces only negligible changes in the calculated HFCCs. Replacing C-PCM with IEF-PCM likewise has little effect on the *A*_iso_ values, with differences within 0.0025 mT (Table S8). The observed trends are robust with respect to further increases in basis-set size and the choice between these two implicit-solvation treatments.

The RSH–PCM and SRSH–PCM results based on gas-phase tuning were compared with the fixed-parameter ωB97X-D functional (Table 1). ωB97X-D yields larger ^13^C MAEs than both RSH–PCM and SRSH–PCM for all three arenes, with similar trends found for ^1^H (Table S9). System-specific tuning appears to improve agreement with experiment, with further improvement achieved through LR dielectric screening in SRSH–PCM.

Figure 3 compares the atom-resolved ^1^H and ^13^C HFCCs of anthracene obtained with RSH–PCM and SRSH–PCM across the α range, using cc-pVDZ and 6-31+G­(d)-J as representative general-purpose and HFCC-specialized basis sets, respectively. For ^1^H HFCCs, the two basis sets yield similar results, consistent with the weak basis-set dependence discussed above. By contrast, dielectric screening generally improves the individual ^1^H HFCCs, with SRSH–PCM yielding closer agreement with experiment than RSH–PCM.

**Figure 3:**
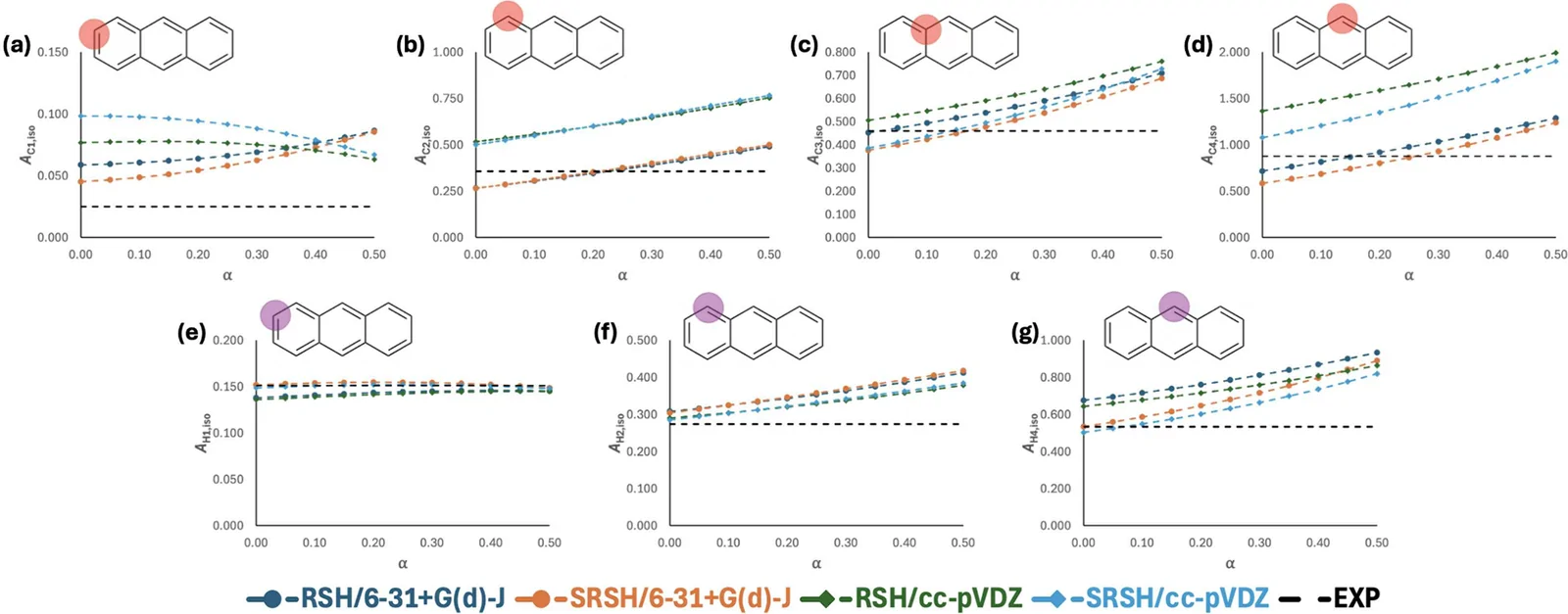
(a–d) ^13^C and (e–g) ^1^H HFCCs of anthracene as a function of α, calculated using RSH–PCM/6-31+G­(d)-J (dark-blue circles), SRSH–PCM/6-31+G­(d)-J (orange circles), RSH–PCM/cc-pVDZ (green diamonds), and SRSH–PCM/cc-pVDZ (light-blue diamonds). Experimental values are shown by the black lines.^70^

The ^13^C HFCCs show stronger basis-set dependence. With cc-pVDZ, both RSH–PCM and SRSH–PCM substantially overestimate three of the four couplings (Figure 3a,b,d), whereas 6-31+G­(d)-J reduces these deviations. With the specialized basis set, the effect of dielectric screening also becomes more apparent: RSH–PCM generally yields larger ^13^C HFCCs than SRSH–PCM across the α range, while the SRSH–PCM values near α = 0.20 provide consistently closer agreement with experiment. The atom-resolved results therefore reinforce the trends obtained from the MAE analysis, highlighting the importance of both an HFCC-specialized basis set and long-range dielectric screening for the ^13^C couplings. Corresponding results for naphthalene and pyrene, together with the individual *A*_iso_ values for all basis sets examined, are presented in SI section 2.1.

Having established the performance of SRSH–PCM for the arene radical-anion benchmark, we next assess its applicability to other molecular systems and across different solvent environments. We first examine experimental ^13^C HFCCs in DME for the semiquinone derivatives shown in Figure 4a: *p*-benzoquinone (PBQ), tetrafluoro-*p*-benzoquinone (TFPBQ), and tetrachloro-*p*-benzoquinone (TCPBQ).^74,75^ These systems extend the analysis to oxygen-containing radicals with F and Cl substituents. Based on the arene analysis, SRSH–PCM calculations were performed using α = 0.20 and the pcH-1 basis set. pcH-1 provides a reliable specialized basis for all atoms in these systems, whereas the ccJ-pVDZ and 6-31+G­(d)-J basis sets considered above are not available for F and Cl.

**Figure 4:**
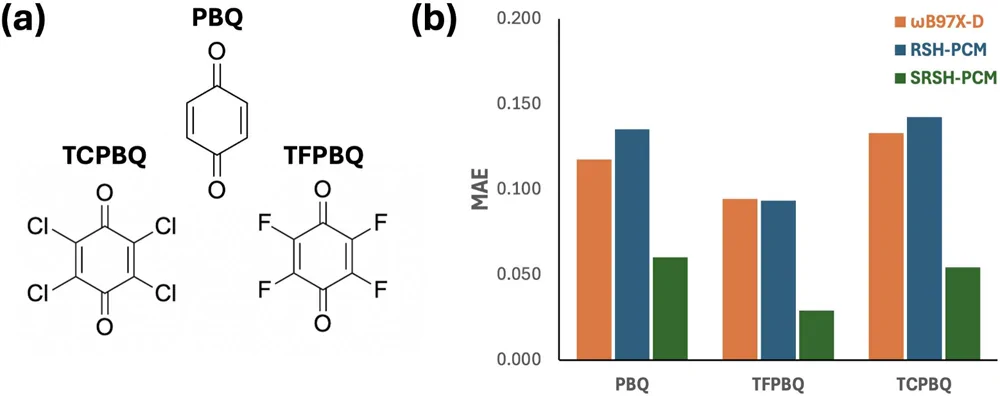
(a) Quinone derivatives (top to bottom, left to right): *p*-benzoquinone (PBQ), tetrafluoro-*p*-benzoquinone (TFPBQ), and tetrachloro-*p*-benzoquinone (TCPBQ). (b) MAEs of the calculated ^13^C HFCCs using ωB97X-D (orange), RSH–PCM (blue), and SRSH–PCM (green). Calculated HFCCs and corresponding experimental values^75^ are presented in Table S17.

We compare SRSH–PCM with RSH–PCM and ωB97X-D, using pcH-1. The geometry of each quinone was optimized at the ωB97X-D/cc-pVDZ level using C-PCM with ε = 7.2 for DME. The gas-phase-tuned ω values obtained at α = 0.20 were used without further tuning in both RSH–PCM and SRSH–PCM; the tuned ω values and calculated HFCCs are reported in Table S17. The resulting MAEs are shown in Figure 4b. SRSH–PCM yields the lowest MAE for each of the three quinones, extending the improvement observed for the arene radicals to the semiquinone derivatives. In particular, the consistent improvement for PBQ and its fluoro- and chloro-substituted derivatives demonstrates that the improved accuracy with α = 0.20 persists without readjusting the SR exact-exchange weight.

We next examine the applicability of SRSH–PCM across solvent environments through the solvent dependence of the ^13^C HFCCs of PBQ. Calculations were performed with ωB97X-D, RSH–PCM, and SRSH–PCM in DME (ε = 7.2), DMSO (ε = 46.7), and H_2_O (ε = 78.4).^36,74,76^ The corresponding MAEs are listed in Table 2, with the tuned ω parameters and individual *A*_iso_ values provided in SI section 2.2. SRSH–PCM remains the most accurate of the three approaches in each solvent. Notably, its MAE remains small from DME to DMSO, decreasing from 0.060 to 0.051 mT despite the substantial increase in dielectric constant. In contrast, all three methods show appreciably larger errors in water, with the SRSH–PCM MAE increasing to 0.145 mT.

**Table 2:** MAEs of the Calculated ^13^C HFCCs of PBQ in DME, DMSO, and Water Using Different Methods, Averaged over the Two Symmetry-Distinct Carbon Environments; Calculated HFCCs and Corresponding Experimental Values^74–76^ Are Presented in Table S18

solvent	ε	ωB97X-D	RSH-PCM	SRSH-PCM
DME	7.2	0.11756	0.13536	0.06037
DMSO	46.7	0.11748	0.13473	0.05090
H_2_O	78.4	0.18160	0.19825	0.14454
H_2_O^a^	78.4	0.14156	0.15227	0.09456

a Includes two explicit H_2_O molecules

The larger errors in water suggest that dielectric screening alone is insufficient to describe the aqueous environment of the semiquinone, where specific hydrogen-bonding interactions with the carbonyl groups are expected to influence the spin density and therefore the HFCCs.^13,16^ To test this interpretation, HFCCs were calculated with two explicit water molecules, one hydrogen bonded to each carbonyl group, while retaining the continuum description of the surrounding aqueous environment. Including these explicit hydrogen-bonding interactions reduces the MAE for all three methods. For SRSH–PCM, the MAE decreases from 0.145 to 0.095 mT and remains the lowest among the three approaches. These results show that SRSH–PCM remains effective across the examined solvent environments, while specific hydrogen-bonding interactions must be represented explicitly when they significantly affect the local spin-density distribution.

In conclusion, the results of this Letter demonstrate that incorporating dielectric screening into range-separated hybrid DFT substantially improves the prediction of condensed-phase HFCCs. Across the examined arene radicals, SRSH–PCM improves ^1^H and ^13^C *A*_iso_ values relative to RSH–PCM, demonstrating the importance of screening the long-range exchange interaction in describing hyperfine interactions in condensed-phase environments. Systematic examination of the basis-set and short-range exact-exchange dependence further shows that this improvement is robust when an HFCC-appropriate basis set and a suitable fraction of short-range exact exchange are employed.

Importantly, the SRSH–PCM protocol established for the arenes transfers beyond both the molecular systems and the single solvent environment modeled in the initial benchmark.

For the semiquinone derivatives, SRSH–PCM again provides the lowest errors among the examined approaches. For PBQ, this improved performance persists across DME, DMSO, and water, spanning a broad range of dielectric environments.

The larger errors observed in water, and their reduction upon inclusion of explicit hydrogen-bonded water molecules, highlight the complementary roles of bulk dielectric screening and specific short-range solute–solvent interactions.

These results establish dielectric screening of long-range exchange as an important component in accurately predicting condensed-phase hyperfine interactions and their response to changes in the molecular environment. The consistent performance observed across nuclei, radical classes, and dielectric environments motivates extending SRSH–PCM to more complex EPR-active systems, including transition-metal molecular qubits,^78,79^ as well as to other magnetic-resonance properties such as *g* tensors^80–82^ and zero-field splittings.^83,84^ Their stronger dependence on orbital energies and energy separations suggests that dielectric screening may lead to even greater improvements in their calculated values. Such applications will be explored in future work.

## Supplementary Material



## Notes

Sketch of atom numbering for calculated HFCCs of the examined arenes; computed MAEs across all basis sets, α values, and methods examined; numerical data for all calculated *A*_iso_ values of the examined arenes; benchmarking of C-PCM results compared to IEF-PCM-computed HFCCs; numerical data for all calculated *A*_iso_ values of the examined benzoquinones; coordinates of all optimized geometries utilized in this study; sample Q-Chem input files for RSH–PCM and SRSH–PCM HFCC calculations (PDF)
