# Supplementary material for: Predictive Description of Condensed-Phase Hyperfine Coupling Constants Relies on Dielectric Screening of Long-Range Exchange
Source: J Phys Chem Lett. 2026 Oct 14;17(43):12217–25. doi: 10.1021/acs.jpclett.6c02938 (PMC13726466; doi:10.1021/acs.jpclett.6c02938)
Supplement: Supplementary file 1 [file jz6c02938_si_001.pdf]

Supporting Information: Predictive Description of Condensed-Phase Hyperfine Coupling Constants Relies on Dielectric Screening of Long-Range Exchange

Slater T. Bakenhaster and Barry D. Dunietz*

Kent State University, Department of Chemistry and Biochemistry, Kent, OH 44242 USA

E-mail: bdunietz@kent.edu

1 Calculated MAEs for Arenes

Table S1: Computed MAEs for HFCCs of naphthalene, anthracene, and pyrene using the cc-pVDZ basis set.

			^{13}C MAE		^{1}H MAE	
System	α	ω	RSH-PCM	SRSH-PCM	RSH-PCM	SRSH-PCM
Naphthalene	0.00	267	0.21604	0.12096	0.05601	0.01750
	0.05	256	0.23645	0.12834	0.06150	0.02714
	0.10	245	0.25828	0.14909	0.06755	0.03724
	0.15	233	0.28074	0.17937	0.07475	0.04779
	0.20	221	0.30347	0.21158	0.08315	0.05883
	0.25	209	0.32651	0.24578	0.09210	0.07034
	0.30	195	0.34980	0.28233	0.10124	0.08241
	0.35	181	0.37461	0.32107	0.11100	0.09497
	0.40	167	0.40097	0.36184	0.12140	0.10802
	0.45	151	0.42826	0.39744	0.13220	0.12163
	0.50	133	0.45685	0.43461	0.14352	0.13579
Anthracene	0.00	234	0.18633	0.12264	0.04673	0.01497
	0.05	225	0.20995	0.13814	0.05436	0.01006
	0.10	215	0.23383	0.15404	0.06216	0.01463
	0.15	204	0.25813	0.17320	0.07022	0.02634
	0.20	193	0.28370	0.20525	0.07901	0.03891
	0.25	182	0.31061	0.23897	0.08855	0.05219
	0.30	170	0.33834	0.27454	0.09860	0.06627
	0.35	157	0.36711	0.31208	0.10928	0.08118
	0.40	145	0.39811	0.35121	0.12123	0.09699
	0.45	129	0.42921	0.39296	0.13333	0.11455
	0.50	114	0.46295	0.43599	0.14692	0.13287
Pyrene	0.00	238	0.18873	0.12258	0.05497	0.01367
	0.05	228	0.21655	0.12465	0.06581	0.01374
	0.10	217	0.24530	0.14369	0.07712	0.02160
	0.15	206	0.27650	0.17098	0.08956	0.03728
	0.20	194	0.30923	0.20018	0.10274	0.05439
	0.25	182	0.34498	0.23937	0.11730	0.07304
	0.30	170	0.38405	0.28783	0.13336	0.09335
	0.35	155	0.42404	0.34134	0.14991	0.11577
	0.40	142	0.47016	0.39896	0.16913	0.13993
	0.45	126	0.51873	0.46250	0.18948	0.16653
	0.50	110	0.57291	0.53099	0.21224	0.19519

Table S2: Computed MAEs for HFCCs of naphthalene, anthracene, and pyrene using the 6-31+G(d) basis set.

			^{13}C MAE		^{1}H MAE	
System	α	ω	RSH-PCM	SRSH-PCM	RSH-PCM	SRSH-PCM
Naphthalene	0.00	258	0.12298	0.10809	0.08386	0.04835
	0.05	248	0.14384	0.11571	0.09233	0.05909
	0.10	237	0.16503	0.12361	0.10102	0.07029
	0.15	226	0.18731	0.13179	0.11024	0.08200
	0.20	214	0.21015	0.14532	0.11978	0.09422
	0.25	202	0.23424	0.17732	0.12991	0.10697
	0.30	189	0.25914	0.20850	0.14045	0.12031
	0.35	175	0.44561	0.24132	0.15142	0.13425
	0.40	160	0.31215	0.27585	0.16304	0.14881
	0.45	145	0.34101	0.31189	0.17535	0.16391
	0.50	128	0.37115	0.34971	0.18819	0.17964
Anthracene	0.00	225	0.11183	0.07838	0.07508	0.02439
	0.05	215	0.12555	0.09382	0.08359	0.03651
	0.10	206	0.14365	0.10978	0.09324	0.04934
	0.15	195	0.16932	0.12623	0.10277	0.06287
	0.20	185	0.19693	0.14324	0.11354	0.07716
	0.25	174	0.22528	0.16526	0.12478	0.09229
	0.30	162	0.25457	0.20123	0.13664	0.10832
	0.35	150	0.45163	0.23911	0.14976	0.12524
	0.40	137	0.31774	0.27906	0.16370	0.14312
	0.45	123	0.35156	0.32107	0.17839	0.16197
	0.50	108	0.38720	0.36499	0.19416	0.18173
Pyrene	0.00	229	0.09454	0.10250	0.08003	0.02703
	0.05	218	0.11463	0.10493	0.09158	0.03203
	0.10	208	0.13672	0.10677	0.10487	0.04809
	0.15	197	0.16729	0.10796	0.11882	0.06553
	0.20	186	0.20106	0.13176	0.13415	0.08452
	0.25	174	0.23676	0.16198	0.15049	0.10526
	0.30	162	0.27572	0.19601	0.16852	0.12786
	0.35	149	0.47588	0.24726	0.18789	0.15258
	0.40	135	0.36271	0.30343	0.20919	0.17961
	0.45	121	0.41268	0.36454	0.23275	0.20891
	0.50	104	0.46633	0.43177	0.25812	0.24102

Table S3: Computed MAEs for HFCCs of naphthalene, anthracene, and pyrene using the 6-31+G(d)-J basis set.

			^{13}C MAE		^{1}H MAE	
System	α	ω	RSH-PCM	SRSH-PCM	RSH-PCM	SRSH-PCM
Naphthalene	0.00	258	0.06490	0.09324	0.06767	0.03149
	0.05	248	0.06184	0.07230	0.07480	0.04228
	0.10	237	0.05885	0.05056	0.08341	0.05351
	0.15	226	0.05600	0.03688	0.09256	0.06522
	0.20	214	0.07746	0.03491	0.10201	0.07742
	0.25	202	0.10336	0.05294	0.11206	0.09014
	0.30	189	0.13030	0.08520	0.12250	0.10340
	0.35	175	0.15848	0.11942	0.13341	0.11723
	0.40	160	0.18812	0.15566	0.14486	0.13163
	0.45	145	0.21974	0.19375	0.15703	0.14652
	0.50	128	0.25296	0.23390	0.16971	0.16199
Anthracene	0.00	225	0.07283	0.12190	0.06298	0.01038
	0.05	215	0.05759	0.09900	0.07150	0.02228
	0.10	206	0.04564	0.07494	0.08113	0.03506
	0.15	196	0.03358	0.04972	0.09104	0.04851
	0.20	184	0.04299	0.03187	0.10096	0.06272
	0.25	174	0.06389	0.02825	0.11251	0.07768
	0.30	163	0.09122	0.05274	0.12461	0.09346
	0.35	151	0.11967	0.08618	0.13737	0.11013
	0.40	138	0.14945	0.12160	0.15096	0.12772
	0.45	123	0.18052	0.15912	0.16531	0.14669
	0.50	108	0.21370	0.19826	0.18106	0.16735
Pyrene	0.00	229	0.05242	0.11083	0.06772	0.01820
	0.05	218	0.05130	0.08440	0.07920	0.02293
	0.10	208	0.05074	0.05574	0.09240	0.03701
	0.15	198	0.06750	0.02786	0.10680	0.05431
	0.20	187	0.09256	0.02871	0.12199	0.07311
	0.25	174	0.11852	0.05168	0.13767	0.09369
	0.30	162	0.14777	0.08640	0.15554	0.11606
	0.35	149	0.18384	0.12959	0.17482	0.14049
	0.40	135	0.22371	0.17762	0.19580	0.16718
	0.45	121	0.26754	0.22988	0.21910	0.19608
	0.50	105	0.31497	0.28703	0.24440	0.22753

Table S4: Computed MAEs for HFCCs of naphthalene, anthracene, and pyrene using the ccJ-pVDZ basis set.

			^{13}C MAE		^{1}H MAE	
System	α	ω	RSH-PCM	SRSH-PCM	RSH-PCM	SRSH-PCM
Naphthalene	0.00	265	0.09824	0.09843	0.05481	0.01482
	0.05	254	0.09425	0.07680	0.06088	0.02528
	0.10	243	0.09035	0.06874	0.06756	0.03620
	0.15	231	0.08651	0.06550	0.07454	0.04760
	0.20	219	0.08507	0.06266	0.08317	0.05950
	0.25	207	0.11120	0.06157	0.09297	0.07189
	0.30	194	0.13837	0.09375	0.10317	0.08481
	0.35	180	0.16679	0.12787	0.11383	0.09826
	0.40	165	0.19666	0.16402	0.12501	0.11226
	0.45	149	0.22820	0.20219	0.13677	0.12679
	0.50	132	0.26165	0.24226	0.14917	0.14178
Anthracene	0.00	233	0.09502	0.13161	0.04488	0.01616
	0.05	223	0.08276	0.10851	0.05285	0.01094
	0.10	213	0.07024	0.08429	0.06147	0.01462
	0.15	203	0.05746	0.06680	0.07078	0.02725
	0.20	192	0.05119	0.05364	0.08044	0.04075
	0.25	181	0.06606	0.04010	0.09091	0.05501
	0.30	169	0.08550	0.04573	0.10193	0.07009
	0.35	156	0.11350	0.07885	0.11363	0.08606
	0.40	142	0.14285	0.11400	0.12615	0.10294
	0.45	128	0.17405	0.15097	0.13987	0.12118
	0.50	112	0.20679	0.18994	0.15448	0.14081
Pyrene	0.00	236	0.07698	0.10176	0.05668	0.01203
	0.05	226	0.07551	0.07572	0.06843	0.01301
	0.10	216	0.07443	0.05481	0.08125	0.02482
	0.15	204	0.08681	0.04851	0.09416	0.04153
	0.20	193	0.11243	0.04936	0.10894	0.05974
	0.25	181	0.13964	0.06950	0.12466	0.07960
	0.30	168	0.16880	0.10465	0.14155	0.10133
	0.35	154	0.20033	0.14373	0.15986	0.12512
	0.40	140	0.24122	0.19257	0.18023	0.15100
	0.45	125	0.28557	0.24591	0.20252	0.17919
	0.50	108	0.33363	0.30423	0.22682	0.20989

Table S5: Computed MAEs for HFCCs of naphthalene, anthracene, and pyrene using the pcH-1 basis set.

			^{13}C MAE		^{1}H MAE	
System	α	ω	RSH-PCM	SRSH-PCM	RSH-PCM	SRSH-PCM
Naphthalene	0.00	264	0.09160	0.09244	0.05787	0.01817
	0.05	253	0.08764	0.07109	0.06398	0.02870
	0.10	242	0.08380	0.06296	0.07070	0.03971
	0.15	229	0.07994	0.05990	0.07738	0.05119
	0.20	219	0.08914	0.05723	0.08685	0.06316
	0.25	206	0.11452	0.06386	0.09653	0.07565
	0.30	193	0.14137	0.09564	0.10684	0.08865
	0.35	180	0.16977	0.12919	0.11776	0.10214
	0.40	165	0.19915	0.16483	0.12903	0.11623
	0.45	150	0.23042	0.20228	0.14099	0.13078
	0.50	132	0.26298	0.24194	0.15337	0.14593
Anthracene	0.00	231	0.08651	0.12410	0.05034	0.01398
	0.05	222	0.07416	0.10105	0.05895	0.00894
	0.10	212	0.06172	0.07697	0.06778	0.02051
	0.15	202	0.04903	0.05987	0.07730	0.03324
	0.20	191	0.05301	0.04685	0.08719	0.04697
	0.25	180	0.06787	0.03487	0.09790	0.06147
	0.30	168	0.09295	0.05195	0.10918	0.07680
	0.35	155	0.12078	0.08494	0.12114	0.09303
	0.40	142	0.15025	0.11978	0.13420	0.11020
	0.45	129	0.18147	0.15639	0.14840	0.12887
	0.50	112	0.21365	0.19532	0.16307	0.14901
Pyrene	0.00	235	0.07217	0.09735	0.05908	0.01058
	0.05	225	0.07077	0.07047	0.07083	0.01527
	0.10	215	0.06977	0.04947	0.08365	0.02703
	0.15	204	0.08870	0.04468	0.09709	0.04376
	0.20	192	0.11337	0.04566	0.11134	0.06198
	0.25	180	0.14030	0.06938	0.12704	0.08186
	0.30	168	0.16977	0.10404	0.14436	0.10350
	0.35	154	0.20448	0.14530	0.16262	0.12727
	0.40	139	0.24416	0.19375	0.18258	0.15328
	0.45	125	0.28832	0.24620	0.20515	0.18131
	0.50	109	0.33595	0.30356	0.22962	0.21186

Table S6: Computed MAEs for HFCCs of naphthalene, anthracene, and pyrene using the pcH-2 basis set.

			^{13}C MAE		^{1}H MAE	
System	α	ω	RSH-PCM	SRSH-PCM	RSH-PCM	SRSH-PCM
Naphthalene	0.00	255	0.07851	0.10842	0.05228	0.01896
	0.05	244	0.07503	0.08740	0.06024	0.02955
	0.10	234	0.07174	0.06546	0.06899	0.04061
	0.15	222	0.06845	0.04805	0.07773	0.05210
	0.20	210	0.06533	0.04588	0.08705	0.06409
	0.25	198	0.08935	0.04414	0.09694	0.07656
	0.30	185	0.11666	0.07329	0.10723	0.08956
	0.35	171	0.14527	0.10765	0.11797	0.10308
	0.40	157	0.17575	0.14396	0.12938	0.11708
	0.45	141	0.20761	0.18251	0.14121	0.13165
	0.50	124	0.24151	0.22306	0.15368	0.14668
Anthracene	0.00	224	0.09161	0.13801	0.04454	0.01508
	0.05	214	0.07481	0.11519	0.05265	0.01074
	0.10	205	0.06261	0.09119	0.06183	0.01910
	0.15	194	0.05040	0.06599	0.07088	0.03207
	0.20	184	0.03777	0.04682	0.08107	0.04573
	0.25	173	0.05077	0.03360	0.09171	0.06016
	0.30	161	0.07425	0.03705	0.10292	0.07541
	0.35	148	0.10259	0.07057	0.11483	0.09154
	0.40	135	0.13271	0.10602	0.12784	0.10848
	0.45	121	0.16445	0.14348	0.14179	0.12629
	0.50	106	0.19806	0.18283	0.15684	0.14489
Pyrene	0.00	227	0.06146	0.12446	0.05595	0.01130
	0.05	217	0.05831	0.09822	0.06780	0.01597
	0.10	207	0.05739	0.06986	0.08071	0.02768
	0.15	196	0.05674	0.04010	0.09426	0.04456
	0.20	184	0.07784	0.03476	0.10863	0.06295
	0.25	173	0.10491	0.04047	0.12494	0.08290
	0.30	160	0.13325	0.07476	0.14194	0.10474
	0.35	147	0.16605	0.11407	0.16076	0.12850
	0.40	133	0.20600	0.16181	0.18123	0.15441
	0.45	118	0.24946	0.21404	0.20364	0.18258
	0.50	102	0.29710	0.27092	0.22832	0.21303

Table S7: Computed MAEs for HFCCs of pyrene using the 6-311+G(d)-J basis set.

			^{13}C MAE		^{1}H MAE	
System	α	ω	RSH-PCM	SRSH-PCM	RSH-PCM	SRSH-PCM
Naphthalene	0.00	257	0.06299	0.08777	0.06180	0.02807
	0.05	247	0.06013	0.06683	0.07014	0.03875
	0.10	236	0.05731	0.04501	0.07870	0.04989
	0.15	224	0.06194	0.03816	0.08755	0.06151
	0.20	213	0.08708	0.03613	0.09723	0.07363
	0.25	200	0.11266	0.05939	0.10704	0.08630
	0.30	187	0.13988	0.09213	0.11750	0.09949
	0.35	173	0.16844	0.12696	0.12844	0.11326
	0.40	159	0.19892	0.16377	0.14006	0.12754
	0.45	143	0.23080	0.20290	0.15213	0.14242
	0.50	126	0.26477	0.24408	0.16487	0.15782
Anthracene	0.00	224	0.06404	0.11679	0.05413	0.01319
	0.05	214	0.05262	0.09378	0.06250	0.01654
	0.10	205	0.04072	0.06952	0.07198	0.02914
	0.15	194	0.03300	0.04401	0.08134	0.04242
	0.20	185	0.04801	0.03014	0.09228	0.05642
	0.25	174	0.07201	0.02763	0.10328	0.07121
	0.30	162	0.09919	0.05787	0.11488	0.08686
	0.35	150	0.12803	0.09192	0.12750	0.10336
	0.40	137	0.15829	0.12808	0.14095	0.12078
	0.45	123	0.19024	0.16632	0.15538	0.13913
	0.50	107	0.22390	0.20668	0.17078	0.15843
Pyrene	0.00	228	0.04909	0.10464	0.06320	0.01659
	0.05	219	0.04840	0.07786	0.07584	0.02129
	0.10	208	0.05091	0.04902	0.08842	0.03422
	0.15	197	0.07409	0.02634	0.10225	0.05141
	0.20	185	0.09865	0.02755	0.11693	0.07016
	0.25	174	0.12630	0.05679	0.13361	0.09054
	0.30	161	0.15899	0.09467	0.15102	0.11287
	0.35	148	0.19656	0.13941	0.17033	0.13720
	0.40	135	0.23774	0.18826	0.19168	0.16363
	0.45	119	0.28145	0.24233	0.21437	0.19273
	0.50	104	0.33052	0.30070	0.24001	0.22390

Table S8: MAE of HFCCs for naphthalene, anthracene, and pyrene with the IEF-PCM solvtion model compared to the reference C-PCM with SRSH-PCM, $\alpha = 0.20$ and the 6-31+G(d)-J basis set.

		IEF-PCM	
Molecule	Property	RSH-PCM	SRSH-PCM
Naphthalene	^{13}C MAE	0.07965	0.03494
	^{13}C ΔMAE	0.00219	0.00003
	^{1}H MAE	0.10161	0.07698
	^{1}H ΔMAE	-0.00040	-0.00045
Anthracene	^{13}C MAE	0.04307	0.03154
	^{13}C ΔMAE	0.00008	-0.00033
	^{1}H MAE	0.10108	0.06229
	^{1}H ΔMAE	0.00012	-0.00044
Pyrene	^{13}C MAE	0.09254	0.02864
	^{13}C ΔMAE	-0.00001	-0.00007
	^{1}H MAE	0.12158	0.07270
	^{1}H ΔMAE	-0.00040	-0.00042

Table S9: MAEs for ^{1}H HFCCs of each arene

	Naphthalene			Anthracene			Pyrene		
Method	6-31+G(d)-J	ccJ-pVDZ	pcH-1	6-31+G(d)-J	ccJ-pVDZ	pcH-1	6-31+G(d)-J	ccJ-pVDZ	pcH-1
ωb97xd	0.06859	0.05852	0.06522	0.07912	0.06419	0.07357	0.08688	0.07930	0.08598
RSH-PCM	0.06767^{a}	0.05481^{a}	0.05709^{a}	0.06298^{a}	0.04488^{a}	0.05034^{a}	0.06772^{a}	0.05668^{a}	0.05908^{a}
SRSH-PCM	0.03149^{a}	0.01482^{a}	0.01808^{a}	0.01038^{a}	0.01094^{b}	0.00894^{b}	0.01820^{a}	0.01203^{a}	0.01058^{a}

^{a} $\alpha = 0.00$, ^{b} $\alpha = 0.05$

2 Calculated A_{iso} Values

2.1 A_{iso} Values of Arenes Across Basis Sets and Exact Exchange Fractions

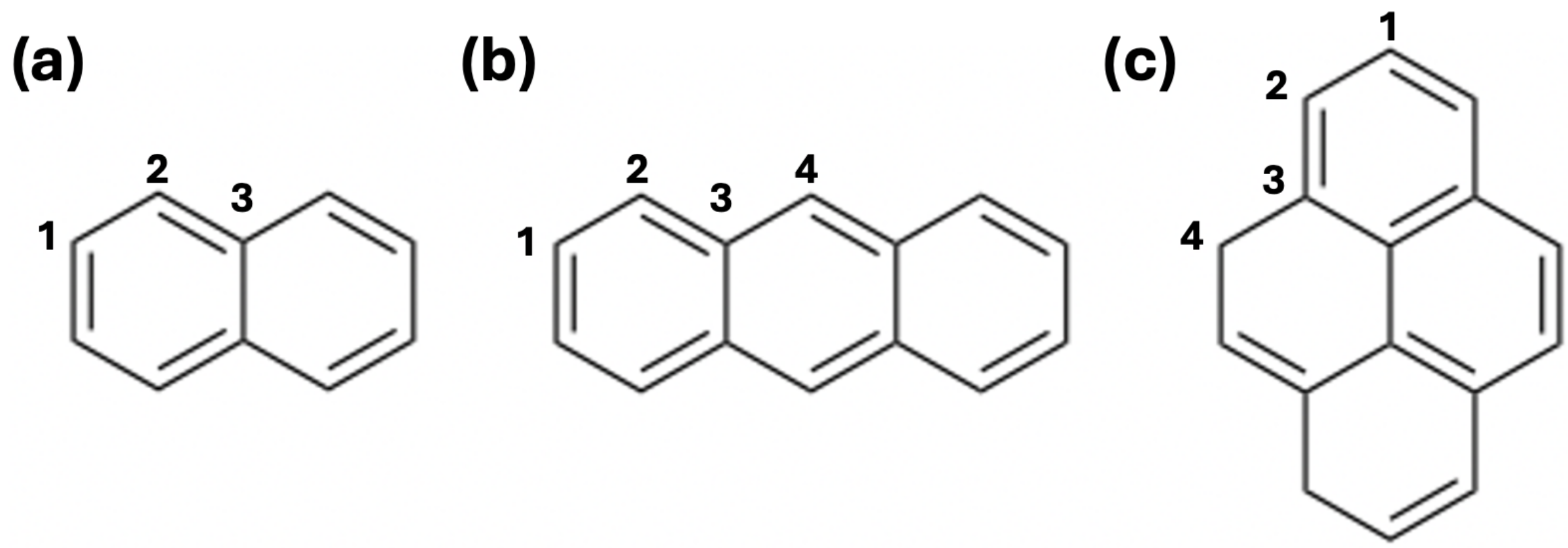

Figure S1: The examined arenes with numbered positions (from left-to-right): naphthalene, anthracene, and pyrene.

		$\alpha = 0$		$\alpha = 0.05$		$\alpha = 0.10$		$\alpha = 0.15$		$\alpha = 0.20$		$\alpha = 0.25$		$\alpha = 0.30$		$\alpha = 0.35$		$\alpha = 0.40$		$\alpha = 0.45$		$\alpha = 0.50$		
System	HFCC	RSH	SRSH	RSH	SRSH	RSH	SRSH	RSH	SRSH	RSH	SRSH	RSH	SRSH	RSH	SRSH	RSH	SRSH	RSH	SRSH	RSH	SRSH	RSH	SRSH	EXP
Naphthalene	$A_{\mathrm{iso,C1}}$	0.0087	0.0522	0.0067	0.0492	0.0042	0.0453	0.0014	0.0404	0.0021	0.0346	0.0063	0.0276	0.0108	0.0195	0.0163	0.0103	0.0228	0.0001	0.0301	0.0119	0.0383	0.0249	0.109
	$A_{\mathrm{iso,C2}}$	1.1528	0.9914	1.1900	1.0396	1.2294	1.0899	1.2697	1.1424	1.3124	1.1972	1.3575	1.2543	1.4032	1.3142	1.4518	1.3767	1.5034	1.4415	1.5568	1.5091	1.6128	1.5793	0.726
	$A_{\mathrm{iso,C3}}$	0.6941	0.5323	0.7160	0.5614	0.7396	0.5926	0.7639	0.6261	0.7901	0.6621	0.8184	0.7007	0.8471	0.7423	0.8783	0.7868	0.9124	0.8342	0.9480	0.8851	0.9861	0.9393	0.573
	$A_{\mathrm{iso,H1}}$	0.1791	0.1900	0.1808	0.1915	0.1823	0.1928	0.1839	0.1939	0.1853	0.1948	0.1865	0.1953	0.1877	0.1955	0.1886	0.1954	0.1893	0.1950	0.1897	0.1942	0.1898	0.1930	0.183
	$A_{\mathrm{iso,H2}}$	0.6031	0.5230	0.6158	0.5408	0.6294	0.5596	0.6436	0.5797	0.6590	0.6009	0.6757	0.6234	0.6928	0.6473	0.7114	0.6725	0.7315	0.6990	0.7527	0.7271	0.7752	0.7566	0.495
Anthracene	$A_{\mathrm{iso,C1}}$	0.0767	0.0982	0.0773	0.0982	0.0776	0.0976	0.0777	0.0963	0.0774	0.0943	0.0765	0.0916	0.0751	0.0881	0.0732	0.0838	0.0705	0.0789	0.0673	0.0732	0.0632	0.0669	0.025
	$A_{\mathrm{iso,C2}}$	0.5163	0.5010	0.5362	0.5250	0.5566	0.5496	0.5776	0.5749	0.5996	0.6008	0.6227	0.6274	0.6466	0.6545	0.6715	0.6821	0.6977	0.7100	0.7248	0.7379	0.7532	0.7658	0.357
	$A_{\mathrm{iso,C3}}$	0.5053	0.3861	0.5255	0.4104	0.5460	0.4365	0.5672	0.4645	0.5899	0.4948	0.6143	0.5273	0.6399	0.5623	0.6668	0.5999	0.6966	0.6396	0.7267	0.6827	0.7602	0.7276	0.459
	$A_{\mathrm{iso,C4}}$	1.3640	1.0764	1.4179	1.1388	1.4721	1.2045	1.5270	1.2741	1.5849	1.3480	1.6460	1.4266	1.7088	1.5102	1.7739	1.5995	1.8447	1.6933	1.9149	1.7950	1.9922	1.9007	0.876
	$A_{\mathrm{iso,H1}}$	0.1359	0.1486	0.1373	0.1498	0.1387	0.1507	0.1401	0.1515	0.1414	0.1520	0.1425	0.1521	0.1435	0.1520	0.1442	0.1515	0.1447	0.1507	0.1450	0.1495	0.1448	0.1480	0.151
	$A_{\mathrm{iso,H2}}$	0.2901	0.2853	0.2971	0.2939	0.3043	0.3029	0.3118	0.3122	0.3198	0.3218	0.3284	0.3318	0.3374	0.3420	0.3468	0.3524	0.3569	0.3630	0.3675	0.3737	0.3786	0.3843	0.274
	$A_{\mathrm{iso,H4}}$	0.6429	0.5028	0.6603	0.5250	0.6779	0.5487	0.6960	0.5743	0.7156	0.6019	0.7368	0.6317	0.7589	0.6638	0.7823	0.6986	0.8084	0.7356	0.8345	0.7764	0.8640	0.8193	0.534
Pyrene	$A_{\mathrm{iso,C1}}$	0.7562	0.5430	0.7943	0.5870	0.8340	0.6352	0.8776	0.6883	0.9238	0.7469	0.9750	0.8115	1.0315	0.8825	1.0897	0.9617	1.1578	1.0477	1.2299	1.1432	1.3110	1.2466	0.600
	$A_{\mathrm{iso,C2}}$	1.1615	0.9355	1.2104	0.9923	1.2607	1.0529	1.3147	1.1180	1.3710	1.1880	1.4319	1.2634	1.4979	1.3448	1.5650	1.4337	1.6416	1.5288	1.7216	1.6328	1.8101	1.7441	0.710
	$A_{\mathrm{iso,C3}}$	0.2815	0.1685	0.2958	0.1847	0.3108	0.2031	0.3277	0.2238	0.3457	0.2473	0.3660	0.2738	0.3890	0.3038	0.4128	0.3381	0.4416	0.3763	0.4725	0.4199	0.5082	0.4682	0.258
	$A_{\mathrm{iso,C4}}$	0.3078	0.3023	0.3177	0.3140	0.3277	0.3257	0.3380	0.3374	0.3484	0.3491	0.3590	0.3607	0.3698	0.3723	0.3806	0.3837	0.3917	0.3950	0.4030	0.4062	0.4143	0.4171	0.184
	$A_{\mathrm{iso,H1}}$	0.1650	0.0854	0.1764	0.0992	0.1884	0.1145	0.2020	0.1317	0.2167	0.1510	0.2333	0.1726	0.2521	0.1968	0.2716	0.2241	0.2950	0.2542	0.3202	0.2879	0.3489	0.3249	0.109
	$A_{\mathrm{iso,H2}}$	0.5741	0.4689	0.5916	0.4905	0.6097	0.5139	0.6295	0.5392	0.6505	0.5668	0.6736	0.5967	0.6989	0.6293	0.7249	0.6652	0.7551	0.7038	0.7869	0.7463	0.8224	0.7921	0.475
	$A_{\mathrm{iso,H4}}$	0.2178	0.2193	0.2215	0.2239	0.2252	0.2284	0.2291	0.2329	0.2330	0.2373	0.2370	0.2417	0.2411	0.2460	0.2452	0.2500	0.2493	0.2538	0.2534	0.2573	0.2573	0.2606	0.208

Table S10: Calculated A_{iso} values for naphthalene, anthracene, and pyrene with the cc-pVDZ basis set.

System	HFCC	$\alpha = 0$		$\alpha = 0.05$		$\alpha = 0.10$		$\alpha = 0.15$		$\alpha = 0.20$		$\alpha = 0.25$		$\alpha = 0.30$		$\alpha = 0.35$		$\alpha = 0.40$		$\alpha = 0.45$		$\alpha = 0.50$		EXP
		RSH	SRSH	RSH	SRSH	RSH	SRSH	RSH	SRSH	RSH	SRSH	RSH	SRSH	RSH	SRSH	RSH	SRSH	RSH	SRSH	RSH	SRSH	RSH	SRSH	
Naphthalene	$A_{\mathrm{iso,C1}}$	0.0083	0.0240	0.0115	0.0202	0.0150	0.0156	0.0192	0.0100	0.0238	0.0035	0.0292	0.0041	0.0352	0.0127	0.0051	0.0225	0.0495	0.0334	0.0583	0.0455	0.0680	0.0586	0.109
	$A_{\mathrm{iso,C2}}$	0.9771	0.8420	1.0182	0.8905	1.0600	0.9410	1.1039	0.9937	1.1491	1.0489	1.1966	1.1065	1.2458	1.1669	1.6101	1.2302	1.3507	1.2964	1.4076	1.3652	1.4670	1.4369	0.726
	$A_{\mathrm{iso,C3}}$	0.5901	0.4497	0.6148	0.4791	0.6401	0.5106	0.6672	0.5443	0.6952	0.5806	0.7253	0.6195	0.7567	0.6613	0.9218	0.7063	0.8252	0.7546	0.8637	0.8059	0.9044	0.8608	0.573
	$A_{\mathrm{iso,H1}}$	0.1895	0.2022	0.1911	0.2036	0.1927	0.2047	0.1942	0.2057	0.1955	0.2063	0.1967	0.2065	0.1977	0.2064	0.1984	0.2059	0.1989	0.2050	0.1990	0.2037	0.1987	0.2020	0.183
	$A_{\mathrm{iso,H2}}$	0.6562	0.5725	0.6715	0.5926	0.6873	0.6139	0.7043	0.6363	0.7220	0.6602	0.7411	0.6854	0.7612	0.7122	0.7825	0.7406	0.8052	0.7706	0.8297	0.8021	0.8557	0.8353	0.495
Anthracene	$A_{\mathrm{iso,C1}}$	0.0525	0.0681	0.0526	0.0675	0.0521	0.0662	0.0515	0.0644	0.0503	0.0619	0.0486	0.0586	0.0465	0.0547	0.0807	0.0501	0.0401	0.0448	0.0359	0.0389	0.0309	0.0325	0.025
	$A_{\mathrm{iso,C2}}$	0.4545	0.4409	0.4759	0.4660	0.4986	0.4920	0.5216	0.5187	0.5459	0.5462	0.5711	0.5745	0.5973	0.6035	0.7830	0.6331	0.6531	0.6632	0.6829	0.6937	0.7138	0.7243	0.357
	$A_{\mathrm{iso,C3}}$	0.4228	0.3255	0.4435	0.3499	0.4663	0.3762	0.4890	0.4045	0.5140	0.4350	0.5401	0.4678	0.5674	0.5031	0.6927	0.5409	0.6280	0.5814	0.6613	0.6244	0.6969	0.6699	0.459
	$A_{\mathrm{iso,C4}}$	1.1621	0.9290	1.2162	0.9906	1.2747	1.0561	1.3322	1.1253	1.3945	1.1989	1.4583	1.2771	1.5241	1.3606	1.9671	1.4493	1.6667	1.5439	1.7432	1.6443	1.8242	1.7504	0.876
	$A_{\mathrm{iso,H1}}$	0.1437	0.1583	0.1453	0.1594	0.1466	0.1601	0.1480	0.1606	0.1491	0.1607	0.1501	0.1606	0.1509	0.1600	0.1513	0.1590	0.1515	0.1577	0.1513	0.1559	0.1508	0.1539	0.151
	$A_{\mathrm{iso,H2}}$	0.3197	0.3164	0.3278	0.3264	0.3366	0.3367	0.3456	0.3475	0.3553	0.3586	0.3655	0.3700	0.3762	0.3818	0.3874	0.3938	0.3993	0.4061	0.4118	0.4184	0.4249	0.4308	0.274
	$A_{\mathrm{iso,H4}}$	0.7062	0.5574	0.7252	0.5828	0.7467	0.6102	0.7677	0.6395	0.7914	0.6712	0.8159	0.7053	0.8416	0.7422	0.8695	0.7819	0.8993	0.8246	0.9310	0.8705	0.9653	0.9195	0.534
Pyrene	$A_{\mathrm{iso,C1}}$	0.6239	0.4539	0.6597	0.4953	0.7007	0.5410	0.7437	0.5913	0.7910	0.6467	0.8414	0.7080	0.8971	0.7757	1.1222	0.8504	1.0231	0.9330	1.0965	1.0234	1.1758	1.1234	0.600
	$A_{\mathrm{iso,C2}}$	0.9508	0.7727	0.9977	0.8265	1.0496	0.8843	1.1033	0.9464	1.1610	1.0131	1.2215	1.0853	1.2869	1.1633	1.6798	1.2479	1.4312	1.3399	1.5130	1.4393	1.6002	1.5477	0.710
	$A_{\mathrm{iso,C3}}$	0.2279	0.1331	0.2412	0.1482	0.2568	0.1654	0.2734	0.1850	0.2919	0.2072	0.3120	0.2325	0.3348	0.2612	0.4025	0.2939	0.3877	0.3311	0.4197	0.3729	0.4549	0.4204	0.258
	$A_{\mathrm{iso,C4}}$	0.2675	0.2603	0.2783	0.2727	0.2895	0.2851	0.3008	0.2977	0.3124	0.3104	0.3241	0.3231	0.3361	0.3359	0.4510	0.3488	0.3608	0.3617	0.3735	0.3746	0.3864	0.3876	0.184
	$A_{\mathrm{iso,H1}}$	0.1766	0.0944	0.1884	0.1095	0.2025	0.1265	0.2175	0.1456	0.2345	0.1669	0.2529	0.1908	0.2738	0.2176	0.2967	0.2476	0.3224	0.2811	0.3515	0.3181	0.3833	0.3594	0.109
	$A_{\mathrm{iso,H2}}$	0.6196	0.5123	0.6382	0.5363	0.6595	0.5623	0.6818	0.5904	0.7062	0.6209	0.7320	0.6542	0.7604	0.6903	0.7909	0.7297	0.8242	0.7728	0.8610	0.8194	0.9005	0.8705	0.475
	$A_{\mathrm{iso,H4}}$	0.2358	0.2372	0.2401	0.2423	0.2446	0.2475	0.2491	0.2526	0.2538	0.2577	0.2585	0.2628	0.2633	0.2677	0.2680	0.2724	0.2730	0.2770	0.2777	0.2813	0.2825	0.2852	0.208

Table S11: Calculated A_{iso} values for naphthalene, anthracene, and pyrene with the 6-31+G(d) basis set.

System	HFCC	$\alpha = 0$		$\alpha = 0.05$		$\alpha = 0.10$		$\alpha = 0.15$		$\alpha = 0.20$		$\alpha = 0.25$		$\alpha = 0.30$		$\alpha = 0.35$		$\alpha = 0.40$		$\alpha = 0.45$		$\alpha = 0.50$		
		RSH	SRSH	RSH	SRSH	RSH	SRSH	RSH	SRSH	RSH	SRSH	RSH	SRSH	RSH	SRSH	RSH	SRSH	RSH	SRSH	RSH	SRSH	RSH	SRSH	EXP
Naphthalene	$A_{\mathrm{iso,C1}}$	0.1588	0.1240	0.1631	0.1290	0.1678	0.1348	0.1730	0.1416	0.1787	0.1493	0.1851	0.1580	0.1921	0.1678	0.1998	0.1787	0.2084	0.1908	0.2181	0.2039	0.2288	0.2182	0.109
	$A_{\mathrm{iso,C2}}$	0.6057	0.5336	0.6433	0.5746	0.6816	0.6171	0.7214	0.6613	0.7623	0.7074	0.8050	0.7554	0.8490	0.8054	0.8945	0.8577	0.9419	0.9122	0.9917	0.9688	1.0435	1.0276	0.726
	$A_{\mathrm{iso,C3}}$	0.5976	0.5007	0.6217	0.5275	0.6463	0.5560	0.6724	0.5864	0.6994	0.6189	0.7280	0.6535	0.7578	0.6904	0.7891	0.7299	0.8220	0.7720	0.8574	0.8166	0.8947	0.8639	0.573
	$A_{\mathrm{iso,H1}}$	0.1817	0.1935	0.1833	0.1950	0.1849	0.1963	0.1863	0.1974	0.1877	0.1981	0.1888	0.1985	0.1898	0.1986	0.1906	0.1982	0.1911	0.1974	0.1912	0.1962	0.1910	0.1946	0.183
	$A_{\mathrm{iso,H2}}$	0.6291	0.5475	0.6443	0.5676	0.6600	0.5887	0.6768	0.6111	0.6944	0.6347	0.7133	0.6597	0.7332	0.6862	0.7542	0.7142	0.7766	0.7438	0.8008	0.7748	0.8264	0.8073	0.495
Anthracene	$A_{\mathrm{iso,C1}}$	0.0587	0.0452	0.0594	0.0466	0.0605	0.0485	0.0620	0.0511	0.0636	0.0542	0.0661	0.0579	0.0690	0.0623	0.0723	0.0672	0.0763	0.0728	0.0809	0.0789	0.0863	0.0854	0.025
	$A_{\mathrm{iso,C2}}$	0.2660	0.2648	0.2850	0.2859	0.3048	0.3075	0.3251	0.3297	0.3460	0.3526	0.3679	0.3760	0.3905	0.4000	0.4140	0.4245	0.4383	0.4494	0.4637	0.4745	0.4900	0.4998	0.357
	$A_{\mathrm{iso,C3}}$	0.4516	0.3766	0.4716	0.3990	0.4934	0.4230	0.5157	0.4487	0.5383	0.4764	0.5634	0.5060	0.5896	0.5376	0.6170	0.5714	0.6458	0.6075	0.6761	0.6461	0.7087	0.6866	0.459
	$A_{\mathrm{iso,C4}}$	0.7168	0.5831	0.7647	0.6328	0.8156	0.6853	0.8672	0.7407	0.9190	0.7996	0.9751	0.8619	1.0328	0.9281	1.0924	0.9986	1.1543	1.0737	1.2184	1.1539	1.2868	1.2382	0.876
	$A_{\mathrm{iso,H1}}$	0.1379	0.1517	0.1395	0.1529	0.1408	0.1538	0.1421	0.1544	0.1434	0.1546	0.1443	0.1546	0.1450	0.1542	0.1456	0.1533	0.1458	0.1521	0.1458	0.1505	0.1452	0.1485	0.151
	$A_{\mathrm{iso,H2}}$	0.3079	0.3041	0.3160	0.3141	0.3247	0.3245	0.3338	0.3352	0.3432	0.3462	0.3533	0.3576	0.3639	0.3692	0.3750	0.3812	0.3867	0.3933	0.3990	0.4054	0.4118	0.4176	0.274
	$A_{\mathrm{iso,H4}}$	0.6759	0.5337	0.6949	0.5589	0.7164	0.5860	0.7384	0.6150	0.7601	0.6463	0.7855	0.6799	0.8120	0.7160	0.8397	0.7549	0.8690	0.7968	0.8997	0.8421	0.9336	0.8900	0.534
Pyrene	$A_{\mathrm{iso,C1}}$	0.6119	0.4773	0.6451	0.5141	0.6824	0.5546	0.7228	0.5991	0.7655	0.6480	0.8098	0.7020	0.8597	0.7613	0.9135	0.8267	0.9721	0.8988	1.0370	0.9774	1.1076	1.0637	0.600
	$A_{\mathrm{iso,C2}}$	0.6095	0.4968	0.6509	0.5409	0.6960	0.5881	0.7437	0.6386	0.7934	0.6929	0.8444	0.7517	0.9003	0.8151	0.9596	0.8840	1.0230	0.9589	1.0921	1.0398	1.1664	1.1278	0.710
	$A_{\mathrm{iso,C3}}$	0.2924	0.2142	0.3058	0.2289	0.3213	0.2456	0.3384	0.2643	0.3565	0.2853	0.3754	0.3091	0.3973	0.3359	0.4212	0.3661	0.4476	0.4003	0.4776	0.4384	0.5108	0.4812	0.258
	$A_{\mathrm{iso,C4}}$	0.1211	0.1204	0.1308	0.1306	0.1408	0.1408	0.1509	0.1511	0.1612	0.1615	0.1716	0.1720	0.1822	0.1827	0.1931	0.1935	0.2041	0.2046	0.2154	0.2159	0.2271	0.2274	0.184
	$A_{\mathrm{iso,H1}}$	0.1720	0.0913	0.1836	0.1062	0.1975	0.1230	0.2131	0.1417	0.2298	0.1627	0.2473	0.1864	0.2679	0.2127	0.2905	0.2423	0.3158	0.2753	0.3444	0.3118	0.3762	0.3522	0.109
	$A_{\mathrm{iso,H2}}$	0.5958	0.4918	0.6143	0.5156	0.6356	0.5415	0.6586	0.5694	0.6828	0.5997	0.7077	0.6327	0.7360	0.6685	0.7663	0.7074	0.7993	0.7499	0.8357	0.7959	0.8752	0.8459	0.475
	$A_{\mathrm{iso,H4}}$	0.2274	0.2282	0.2317	0.2334	0.2362	0.2386	0.2407	0.2438	0.2453	0.2490	0.2500	0.2540	0.2548	0.2590	0.2596	0.2637	0.2644	0.2683	0.2691	0.2726	0.2738	0.2766	0.208

Table S12: Calculated A_{iso} values for naphthalene, anthracene, and pyrene with the 6-31+G(d)-J basis set.

		$\alpha = 0$		$\alpha = 0.05$		$\alpha = 0.10$		$\alpha = 0.15$		$\alpha = 0.20$		$\alpha = 0.25$		$\alpha = 0.30$		$\alpha = 0.35$		$\alpha = 0.40$		$\alpha = 0.45$		$\alpha = 0.50$		
System	HFCC	RSH	SRSH	RSH	SRSH	RSH	SRSH	RSH	SRSH	RSH	SRSH	RSH	SRSH	RSH	SRSH	RSH	SRSH	RSH	SRSH	RSH	SRSH	RSH	SRSH	EXP
	$A_{\mathrm{iso,C1}}$	0.1833	0.1457	0.1862	0.1494	0.1896	0.1539	0.1933	0.1592	0.1978	0.1656	0.2029	0.1731	0.2086	0.1816	0.2151	0.1913	0.2223	0.2023	0.2305	0.2145	0.2398	0.2278	0.109
	$A_{\mathrm{iso,C2}}$	0.5675	0.5007	0.6059	0.5426	0.6458	0.5863	0.6867	0.6317	0.7294	0.6789	0.7738	0.7280	0.8195	0.7791	0.8669	0.8324	0.9160	0.8879	0.9673	0.9456	1.0208	1.0053	0.726
Naphthalene	$A_{\mathrm{iso,C3}}$	0.6350	0.5398	0.6585	0.5663	0.6833	0.5947	0.7089	0.6249	0.7361	0.6572	0.7649	0.6917	0.7950	0.7285	0.8264	0.7679	0.8596	0.8099	0.8948	0.8545	0.9323	0.9016	0.573
	$A_{\mathrm{iso,H1}}$	0.1773	0.1884	0.1790	0.1902	0.1807	0.1917	0.1824	0.1930	0.1839	0.1940	0.1853	0.1947	0.1865	0.1951	0.1875	0.1950	0.1883	0.1946	0.1888	0.1937	0.1889	0.1924	0.183
	$A_{\mathrm{iso,H2}}$	0.5989	0.5192	0.6128	0.5384	0.6278	0.5587	0.6435	0.5802	0.6604	0.6030	0.6787	0.6271	0.6978	0.6525	0.7181	0.6795	0.7397	0.7080	0.7628	0.7379	0.7875	0.7691	0.495
	$A_{\mathrm{iso,C1}}$	0.0698	0.0558	0.0694	0.0560	0.0694	0.0567	0.0698	0.0580	0.0704	0.0599	0.0716	0.0624	0.0733	0.0655	0.0754	0.0694	0.0781	0.0738	0.0816	0.0789	0.0858	0.0844	0.025
	$A_{\mathrm{iso,C2}}$	0.2390	0.2380	0.2583	0.2591	0.2782	0.2809	0.2989	0.3032	0.3201	0.3262	0.3422	0.3497	0.3650	0.3739	0.3887	0.3985	0.4133	0.4236	0.4389	0.4490	0.4655	0.4745	0.357
	$A_{\mathrm{iso,C3}}$	0.4829	0.4044	0.5026	0.4262	0.5234	0.4496	0.5454	0.4748	0.5681	0.5019	0.5923	0.5309	0.6175	0.5621	0.6440	0.5956	0.6720	0.6315	0.7021	0.6695	0.7339	0.7097	0.459
Anthracene	$A_{\mathrm{iso,C4}}$	0.6826	0.5540	0.7317	0.6036	0.7826	0.6560	0.8354	0.7114	0.8893	0.7700	0.9455	0.8322	1.0032	0.8983	1.0629	0.9689	1.1250	1.0440	1.1906	1.1235	1.2589	1.2080	0.876
	$A_{\mathrm{iso,H1}}$	0.1354	0.1482	0.1371	0.1496	0.1387	0.1508	0.1401	0.1517	0.1415	0.1523	0.1427	0.1526	0.1438	0.1526	0.1447	0.1521	0.1453	0.1513	0.1455	0.1502	0.1454	0.1486	0.151
	$A_{\mathrm{iso,H2}}$	0.2900	0.2845	0.2974	0.2939	0.3054	0.3037	0.3138	0.3138	0.3226	0.3243	0.3320	0.3351	0.3418	0.3462	0.3522	0.3576	0.3632	0.3691	0.3747	0.3808	0.3869	0.3924	0.274
	$A_{\mathrm{iso,H4}}$	0.6371	0.4989	0.6552	0.5225	0.6747	0.5479	0.6956	0.5753	0.7172	0.6047	0.7405	0.6363	0.7648	0.6705	0.7904	0.7074	0.8176	0.7474	0.8474	0.7899	0.8790	0.8356	0.534
	$A_{\mathrm{iso,C1}}$	0.6502	0.5093	0.6852	0.5464	0.7230	0.5871	0.7615	0.6319	0.8049	0.6812	0.8510	0.7356	0.9005	0.7956	0.9540	0.8620	1.0134	0.9348	1.0783	1.0148	1.1492	1.1026	0.600
	$A_{\mathrm{iso,C2}}$	0.5975	0.4849	0.6420	0.5304	0.6891	0.5790	0.7368	0.6309	0.7886	0.6868	0.8428	0.7469	0.8999	0.8120	0.9605	0.8826	1.0262	0.9590	1.0967	1.0419	1.1725	1.1319	0.710
	$A_{\mathrm{iso,C3}}$	0.3253	0.2453	0.3397	0.2602	0.3554	0.2769	0.3714	0.2957	0.3899	0.3168	0.4096	0.3407	0.4312	0.3676	0.4548	0.3981	0.4815	0.4324	0.5112	0.4709	0.5443	0.5141	0.258
Pyrene	$A_{\mathrm{iso,C4}}$	0.1062	0.1055	0.1168	0.1165	0.1276	0.1275	0.1384	0.1386	0.1496	0.1498	0.1608	0.1611	0.1723	0.1726	0.1840	0.1842	0.1959	0.1960	0.2081	0.2081	0.2205	0.2204	0.184
	$A_{\mathrm{iso,H1}}$	0.1699	0.0892	0.1820	0.1036	0.1955	0.1197	0.2092	0.1378	0.2255	0.1580	0.2431	0.1808	0.2625	0.2063	0.2839	0.2351	0.3083	0.2670	0.3356	0.3025	0.3660	0.3420	0.109
	$A_{\mathrm{iso,H2}}$	0.5740	0.4695	0.5930	0.4927	0.6136	0.5177	0.6343	0.5447	0.6579	0.5740	0.6829	0.6059	0.7097	0.6407	0.7386	0.6787	0.7706	0.7200	0.8055	0.7649	0.8435	0.8138	0.475
	$A_{\mathrm{iso,H4}}$	0.2182	0.2189	0.2223	0.2240	0.2266	0.2291	0.2309	0.2341	0.2354	0.2392	0.2399	0.2441	0.2445	0.2489	0.2492	0.2536	0.2538	0.2580	0.2584	0.2621	0.2630	0.2659	0.208

Table S13: Calculated A_{iso} values for naphthalene, anthracene, and pyrene with the ccJ-pVDZ basis set.

System	HFCC	$\alpha = 0$		$\alpha = 0.05$		$\alpha = 0.10$		$\alpha = 0.15$		$\alpha = 0.20$		$\alpha = 0.25$		$\alpha = 0.30$		$\alpha = 0.35$		$\alpha = 0.40$		$\alpha = 0.45$		$\alpha = 0.50$		EXP
		RSH	SRSH	RSH	SRSH	RSH	SRSH	RSH	SRSH	RSH	SRSH	RSH	SRSH	RSH	SRSH	RSH	SRSH	RSH	SRSH	RSH	SRSH	RSH	SRSH	
Naphthalene	$A_{\mathrm{iso,C1}}$	0.1799	0.1421	0.1828	0.1459	0.1862	0.1505	0.1898	0.1561	0.1947	0.1627	0.1997	0.1703	0.2056	0.1790	0.2123	0.1887	0.2196	0.1998	0.2280	0.2120	0.2372	0.2255	0.109
	$A_{\mathrm{iso,C2}}$	0.5839	0.5151	0.6220	0.5566	0.6617	0.5997	0.7016	0.6445	0.7449	0.6909	0.7882	0.7393	0.8333	0.7896	0.8803	0.8418	0.9286	0.8964	0.9791	0.9529	1.0312	1.0118	0.726
	$A_{\mathrm{iso,C3}}$	0.6348	0.5396	0.6582	0.5659	0.6828	0.5941	0.7076	0.6240	0.7357	0.6559	0.7636	0.6900	0.7932	0.7264	0.8247	0.7650	0.8573	0.8063	0.8922	0.8500	0.9286	0.8966	0.573
	$A_{\mathrm{iso,H1}}$	0.1773	0.1888	0.1791	0.1906	0.1808	0.1921	0.1827	0.1935	0.1841	0.1945	0.1856	0.1952	0.1868	0.1956	0.1879	0.1956	0.1887	0.1952	0.1892	0.1944	0.1894	0.1932	0.183
	$A_{\mathrm{iso,H2}}$	0.6050	0.5256	0.6191	0.5448	0.6342	0.5653	0.6494	0.5869	0.6676	0.6098	0.6855	0.6341	0.7048	0.6597	0.7257	0.6867	0.7473	0.7152	0.7708	0.7451	0.7953	0.7767	0.495
Anthracene	$A_{\mathrm{iso,C1}}$	0.0673	0.0536	0.0672	0.0540	0.0673	0.0550	0.0679	0.0566	0.0687	0.0587	0.0701	0.0615	0.0719	0.0649	0.0743	0.0690	0.0773	0.0736	0.0810	0.0788	0.0853	0.0846	0.025
	$A_{\mathrm{iso,C2}}$	0.2510	0.2490	0.2704	0.2700	0.2902	0.2916	0.3108	0.3138	0.3318	0.3365	0.3537	0.3598	0.3763	0.3837	0.3997	0.4081	0.4240	0.4329	0.4492	0.4579	0.4754	0.4830	0.357
	$A_{\mathrm{iso,C3}}$	0.4830	0.4048	0.5033	0.4267	0.5240	0.4501	0.5459	0.4753	0.5686	0.5023	0.5926	0.5312	0.6177	0.5622	0.6440	0.5956	0.6721	0.6310	0.7023	0.6685	0.7335	0.7087	0.459
	$A_{\mathrm{iso,C4}}$	0.7024	0.5704	0.7524	0.6201	0.8032	0.6724	0.8559	0.7276	0.9096	0.7861	0.9655	0.8480	1.0229	0.9139	1.0822	0.9842	1.1446	1.0587	1.2103	1.1374	1.2774	1.2220	0.876
	$A_{\mathrm{iso,H1}}$	0.1349	0.1481	0.1365	0.1495	0.1381	0.1506	0.1395	0.1515	0.1409	0.1521	0.1421	0.1523	0.1432	0.1522	0.1440	0.1517	0.1445	0.1509	0.1447	0.1497	0.1446	0.1480	0.151
	$A_{\mathrm{iso,H2}}$	0.2930	0.2893	0.3009	0.2988	0.3089	0.3087	0.3175	0.3189	0.3265	0.3295	0.3360	0.3404	0.3460	0.3516	0.3565	0.3630	0.3676	0.3745	0.3793	0.3862	0.3916	0.3978	0.274
	$A_{\mathrm{iso,H4}}$	0.6499	0.5102	0.6695	0.5345	0.6895	0.5604	0.7109	0.5883	0.7330	0.6184	0.7569	0.6507	0.7818	0.6856	0.8080	0.7234	0.8365	0.7639	0.8676	0.8071	0.8993	0.8542	0.534
Pyrene	$A_{\mathrm{iso,C1}}$	0.6550	0.5124	0.6897	0.5494	0.7273	0.5900	0.7668	0.6344	0.8086	0.6834	0.8543	0.7373	0.9043	0.7966	0.9572	0.8623	1.0148	0.9347	1.0797	1.0135	1.1502	1.0999	0.600
	$A_{\mathrm{iso,C2}}$	0.6103	0.4951	0.6545	0.5402	0.7011	0.5882	0.7493	0.6395	0.7994	0.6945	0.8529	0.7539	0.9101	0.8178	0.9696	0.8874	1.0333	0.9630	1.1034	1.0444	1.1784	1.1328	0.710
	$A_{\mathrm{iso,C3}}$	0.3232	0.2426	0.3374	0.2574	0.3530	0.2740	0.3695	0.2926	0.3872	0.3136	0.4067	0.3372	0.4286	0.3638	0.4519	0.3940	0.4777	0.4281	0.5075	0.4660	0.5405	0.5085	0.258
	$A_{\mathrm{iso,C4}}$	0.1151	0.1125	0.1255	0.1232	0.1361	0.1340	0.1468	0.1449	0.1577	0.1558	0.1687	0.1669	0.1799	0.1781	0.1912	0.1895	0.2028	0.2011	0.2146	0.2129	0.2268	0.2251	0.184
	$A_{\mathrm{iso,H1}}$	0.1707	0.0892	0.1826	0.1035	0.1960	0.1196	0.2103	0.1375	0.2257	0.1577	0.2432	0.1803	0.2629	0.2056	0.2841	0.2342	0.3078	0.2662	0.3354	0.3014	0.3658	0.3404	0.109
	$A_{\mathrm{iso,H2}}$	0.5804	0.4752	0.5995	0.4984	0.6203	0.5236	0.6419	0.5507	0.6648	0.5802	0.6898	0.6123	0.7174	0.6471	0.7463	0.6852	0.7778	0.7269	0.8133	0.7717	0.8517	0.8205	0.475
	$A_{\mathrm{iso,H4}}$	0.2181	0.2198	0.2223	0.2249	0.2267	0.2300	0.2311	0.2350	0.2356	0.2401	0.2401	0.2450	0.2447	0.2498	0.2494	0.2544	0.2542	0.2588	0.2588	0.2629	0.2633	0.2666	0.208

Table S14: Calculated A_{iso} values for naphthalene, anthracene, and pyrene with the pcH-1 basis set.

System	HFCC	α = 0		α = 0.05		α = 0.10		α = 0.15		α = 0.20		α = 0.25		α = 0.30		α = 0.35		α = 0.40		α = 0.45		α = 0.50		
		RSH	SRSH	RSH	SRSH	RSH	SRSH	RSH	SRSH	RSH	SRSH	RSH	SRSH	RSH	SRSH	RSH	SRSH	RSH	SRSH	RSH	SRSH	RSH	SRSH	EXP
Naphthalene	$A_{\mathrm{iso,C1}}$	0.1642	0.1294	0.1679	0.1339	0.1723	0.1393	0.1769	0.1456	0.1821	0.1529	0.1882	0.1612	0.1948	0.1706	0.2022	0.1812	0.2106	0.1928	0.2198	0.2058	0.2302	0.2198	0.109
	$A_{\mathrm{iso,C2}}$	0.5634	0.4986	0.6010	0.5395	0.6408	0.5822	0.6809	0.6266	0.7227	0.6729	0.7664	0.7212	0.8115	0.7716	0.8582	0.8243	0.9072	0.8792	0.9580	0.9366	1.0113	0.9961	0.726
	$A_{\mathrm{iso,C3}}$	0.5907	0.4956	0.6142	0.5222	0.6397	0.5507	0.6653	0.5811	0.6926	0.6137	0.7215	0.6484	0.7517	0.6856	0.7834	0.7255	0.8174	0.7679	0.8530	0.8132	0.8910	0.8612	0.573
	$A_{\mathrm{iso,H1}}$	0.1832	0.1928	0.1850	0.1946	0.1865	0.1962	0.1881	0.1974	0.1896	0.1984	0.1909	0.1991	0.1920	0.1994	0.1929	0.1993	0.1935	0.1988	0.1938	0.1979	0.1938	0.1967	0.183
	$A_{\mathrm{iso,H2}}$	0.5993	0.5231	0.6135	0.5425	0.6294	0.5631	0.6453	0.5848	0.6625	0.6078	0.6810	0.6321	0.7005	0.6577	0.7210	0.6849	0.7433	0.7133	0.7666	0.7434	0.7916	0.7747	0.495
Anthracene	$A_{\mathrm{iso,C1}}$	0.0621	0.0487	0.0624	0.0496	0.0631	0.0511	0.0640	0.0532	0.0655	0.0559	0.0674	0.0591	0.0698	0.0630	0.0726	0.0675	0.0762	0.0726	0.0805	0.0783	0.0855	0.0844	0.025
	$A_{\mathrm{iso,C2}}$	0.2458	0.2448	0.2651	0.2659	0.2854	0.2876	0.3059	0.3099	0.3275	0.3329	0.3497	0.3566	0.3728	0.3809	0.3969	0.4059	0.4219	0.4313	0.4480	0.4572	0.4751	0.4833	0.357
	$A_{\mathrm{iso,C3}}$	0.4490	0.3747	0.4690	0.3968	0.4907	0.4206	0.5125	0.4462	0.5362	0.4737	0.5608	0.5032	0.5865	0.5349	0.6136	0.5690	0.6428	0.6053	0.6737	0.6439	0.7068	0.6848	0.459
	$A_{\mathrm{iso,C4}}$	0.6678	0.5441	0.7160	0.5932	0.7670	0.6451	0.8180	0.7001	0.8721	0.7584	0.9276	0.8203	0.9849	0.8864	1.0442	0.9569	1.1069	1.0319	1.1726	1.1116	1.2419	1.1958	0.876
	$A_{\mathrm{iso,H1}}$	0.1397	0.1516	0.1414	0.1530	0.1430	0.1542	0.1445	0.1551	0.1458	0.1556	0.1470	0.1559	0.1480	0.1558	0.1488	0.1553	0.1493	0.1545	0.1494	0.1533	0.1491	0.1518	0.151
	$A_{\mathrm{iso,H2}}$	0.2924	0.2887	0.3002	0.2983	0.3086	0.3082	0.3172	0.3185	0.3264	0.3291	0.3361	0.3401	0.3462	0.3513	0.3569	0.3627	0.3682	0.3743	0.3801	0.3860	0.3925	0.3977	0.274
	$A_{\mathrm{iso,H4}}$	0.6380	0.5040	0.6562	0.5281	0.6768	0.5539	0.6970	0.5816	0.7196	0.6115	0.7430	0.6436	0.7675	0.6782	0.7934	0.7156	0.8216	0.7556	0.8517	0.7985	0.8842	0.8442	0.534
Pyrene	$A_{\mathrm{iso,C1}}$	0.5963	0.4674	0.6303	0.5037	0.6672	0.5435	0.7060	0.5873	0.7471	0.6355	0.7933	0.6886	0.8418	0.7473	0.8953	0.8119	0.9535	0.8832	1.0173	0.9615	1.0879	1.0470	0.600
	$A_{\mathrm{iso,C2}}$	0.5692	0.4650	0.6119	0.5088	0.6571	0.5556	0.7039	0.6057	0.7528	0.6597	0.8061	0.7179	0.8613	0.7810	0.9210	0.8495	0.9847	0.9239	1.0535	1.0048	1.1283	1.0924	0.710
	$A_{\mathrm{iso,C3}}$	0.2865	0.2110	0.3003	0.2254	0.3155	0.2416	0.3315	0.2599	0.3487	0.2806	0.3686	0.3038	0.3894	0.3302	0.4130	0.3600	0.4391	0.3936	0.4683	0.4314	0.5013	0.4736	0.258
	$A_{\mathrm{iso,C4}}$	0.1113	0.1108	0.1215	0.1213	0.1320	0.1319	0.1426	0.1426	0.1534	0.1534	0.1644	0.1644	0.1756	0.1755	0.1870	0.1869	0.1987	0.1985	0.2106	0.2104	0.2229	0.2227	0.184
	$A_{\mathrm{iso,H1}}$	0.1645	0.0887	0.1766	0.1032	0.1902	0.1195	0.2047	0.1377	0.2204	0.1582	0.2388	0.1810	0.2582	0.2067	0.2803	0.2353	0.3049	0.2673	0.3323	0.3027	0.3632	0.3417	0.109
	$A_{\mathrm{iso,H2}}$	0.5752	0.4758	0.5942	0.4992	0.6150	0.5245	0.6366	0.5517	0.6595	0.5813	0.6853	0.6133	0.7122	0.6483	0.7418	0.6862	0.7739	0.7275	0.8090	0.7724	0.8475	0.8208	0.475
	$A_{\mathrm{iso,H4}}$	0.2202	0.2208	0.2245	0.2259	0.2289	0.2311	0.2334	0.2362	0.2380	0.2413	0.2427	0.2463	0.2474	0.2512	0.2522	0.2559	0.2569	0.2604	0.2616	0.2646	0.2662	0.2686	0.208

Table S15: Calculated A_{iso} values for naphthalene, anthracene, and pyrene with the pcH-2 basis set.

System	HFCC	$\alpha = 0$		$\alpha = 0.05$		$\alpha = 0.10$		$\alpha = 0.15$		$\alpha = 0.20$		$\alpha = 0.25$		$\alpha = 0.30$		$\alpha = 0.35$		$\alpha = 0.40$		$\alpha = 0.45$		$\alpha = 0.50$		EXP
		RSH	SRSH	RSH	SRSH	RSH	SRSH	RSH	SRSH	RSH	SRSH	RSH	SRSH	RSH	SRSH	RSH	SRSH	RSH	SRSH	RSH	SRSH	RSH	SRSH	
Naphthalene	$A_{\text{iso,C1}}$	0.1560	0.1240	0.1604	0.1290	0.1652	0.1349	0.1703	0.1416	0.1763	0.1493	0.1826	0.1580	0.1898	0.1677	0.1976	0.1786	0.2065	0.1905	0.2161	0.2036	0.2269	0.2178	0.109
	$A_{\text{iso,C2}}$	0.6231	0.5400	0.6602	0.5809	0.6981	0.6236	0.7371	0.6681	0.7787	0.7146	0.8209	0.7634	0.8652	0.8143	0.9114	0.8677	0.9599	0.9235	1.0103	0.9819	1.0633	1.0427	0.726
	$A_{\text{iso,C3}}$	0.6121	0.5106	0.6362	0.5376	0.6609	0.5663	0.6864	0.5970	0.7143	0.6297	0.7425	0.6648	0.7726	0.7024	0.8043	0.7426	0.8383	0.7854	0.8739	0.8312	0.9121	0.8798	0.573
	$A_{\text{iso,H1}}$	0.1845	0.1956	0.1861	0.1972	0.1876	0.1986	0.1892	0.1997	0.1905	0.2006	0.1918	0.2011	0.1928	0.2012	0.1937	0.2010	0.1942	0.2003	0.1944	0.1992	0.1942	0.1977	0.183
	$A_{\text{iso,H2}}$	0.6171	0.5386	0.6322	0.5583	0.6478	0.5792	0.6639	0.6013	0.6820	0.6247	0.7003	0.6495	0.7202	0.6758	0.7412	0.7036	0.7640	0.7327	0.7879	0.7636	0.8135	0.7959	0.495
Anthracene	$A_{\text{iso,C1}}$	0.0531	0.0421	0.0538	0.0434	0.0549	0.0453	0.0562	0.0476	0.0582	0.0505	0.0604	0.0540	0.0631	0.0581	0.0664	0.0628	0.0703	0.0680	0.0748	0.0738	0.0800	0.080	0.025
	$A_{\text{iso,C2}}$	0.2722	0.2665	0.2911	0.2876	0.3110	0.3094	0.3313	0.3318	0.3528	0.3550	0.3748	0.3789	0.3977	0.4034	0.4216	0.4286	0.4466	0.4543	0.4726	0.4805	0.4998	0.507	0.357
	$A_{\text{iso,C3}}$	0.4619	0.3846	0.4820	0.4070	0.5038	0.4312	0.5257	0.4572	0.5502	0.4850	0.5750	0.5149	0.6009	0.5470	0.6286	0.5814	0.6579	0.6181	0.6891	0.6572	0.7220	0.699	0.459
	$A_{\text{iso,C4}}$	0.7357	0.5909	0.7832	0.6407	0.8338	0.6936	0.8843	0.7496	0.9395	0.8090	0.9949	0.8723	1.0521	0.9399	1.1124	1.0119	1.1754	1.0888	1.2415	1.1707	1.3108	1.258	0.876
	$A_{\text{iso,H1}}$	0.1405	0.1538	0.1422	0.1550	0.1436	0.1560	0.1451	0.1568	0.1462	0.1572	0.1473	0.1573	0.1482	0.1570	0.1488	0.1564	0.1492	0.1553	0.1492	0.1539	0.1489	0.152	0.151
	$A_{\text{iso,H2}}$	0.3008	0.2979	0.3088	0.3077	0.3174	0.3179	0.3262	0.3284	0.3359	0.3393	0.3458	0.3505	0.3562	0.3620	0.3673	0.3738	0.3789	0.3857	0.3911	0.3977	0.4039	0.410	0.274
	$A_{\text{iso,H4}}$	0.6592	0.5211	0.6779	0.5459	0.6991	0.5725	0.7199	0.6011	0.7441	0.6318	0.7683	0.6648	0.7936	0.7006	0.8211	0.7390	0.8502	0.7803	0.8813	0.8248	0.9143	0.872	0.534
Pyrene	$A_{\text{iso,C1}}$	0.6244	0.4855	0.6604	0.5230	0.6967	0.5638	0.7363	0.6088	0.7783	0.6584	0.8255	0.7130	0.8750	0.7735	0.9298	0.8401	0.9903	0.9132	1.0550	0.9944	1.1280	1.0823	0.600
	$A_{\text{iso,C2}}$	0.6288	0.5063	0.6724	0.5509	0.7163	0.5984	0.7631	0.6496	0.8120	0.7049	0.8656	0.7645	0.9212	0.8295	0.9816	0.9000	1.0471	0.9765	1.1163	1.0606	1.1932	1.1508	0.710
	$A_{\text{iso,C3}}$	0.2946	0.2163	0.3095	0.2312	0.3244	0.2478	0.3408	0.2666	0.3585	0.2877	0.3788	0.3116	0.4002	0.3386	0.4243	0.3691	0.4516	0.4033	0.4810	0.4424	0.5152	0.4854	0.258
	$A_{\text{iso,C4}}$	0.1300	0.1252	0.1398	0.1355	0.1497	0.1459	0.1598	0.1564	0.1701	0.1671	0.1807	0.1780	0.1915	0.1891	0.2026	0.2004	0.2139	0.2120	0.2256	0.2239	0.2377	0.2362	0.184
	$A_{\text{iso,H1}}$	0.1681	0.0898	0.1813	0.1047	0.1944	0.1213	0.2092	0.1399	0.2253	0.1609	0.2441	0.1843	0.2641	0.2106	0.2868	0.2400	0.3125	0.2727	0.3403	0.3094	0.3725	0.3494	0.109
	$A_{\text{iso,H2}}$	0.5891	0.4878	0.6095	0.5116	0.6297	0.5373	0.6518	0.5651	0.6752	0.5952	0.7017	0.6280	0.7292	0.6637	0.7596	0.7026	0.7932	0.7447	0.8286	0.7911	0.8687	0.8406	0.475
	$A_{\text{iso,H4}}$	0.2244	0.2258	0.2287	0.2310	0.2331	0.2361	0.2377	0.2413	0.2423	0.2464	0.2470	0.2514	0.2518	0.2563	0.2566	0.2610	0.2614	0.2655	0.2662	0.2697	0.2708	0.2736	0.208

Table S16: Calculated A_{iso} values for naphthalene, anthracene, and pyrene with the 6-311+G(d)-J basis set.

2.2 A_{iso} Values of PBQ, TFPBQ, and TCPBQ

Table S17: Computed A_{iso} values for PBQ, TFPBQ, and TCPBQ in DME.

Molecule	ω	HFCC	ωB97X-D	RSH-PCM	SRSH-PCM	EXP
PBQ	303	$A_{\text{C=O,iso}}$	0.36893	0.42171	0.31102	0.221
		$A_{\text{C-C,iso}}$	0.09918	0.08201	0.04272	0.012
TFPBQ	273	$A_{\text{C=O,iso}}$	0.34673	0.37058	0.26131	0.219
		$A_{\text{C-C,iso}}$	0.14130	0.11527	0.09575	0.080
TCPBQ	220	$A_{\text{C=O,iso}}$	0.39015	0.39937	0.31141	0.257
		$A_{\text{C-C,iso}}$	0.05871	0.02529	0.01300	-

Table S18: Computed A_{iso} values for PBQ in DME, DMSO, and H_2O.

Solvent	ω	HFCC	ωB97X-D	RSH-PCM	SRSH-PCM	EXP
DME	303	$A_{\text{C=O,iso}}$	0.36893	0.42171	0.31102	0.221
		$A_{\text{C-C,iso}}$	0.09918	0.08201	0.04272	0.012
DMSO	304	$A_{\text{C=O,iso}}$	0.35504	0.40563	0.28470	0.213
		$A_{\text{C-C,iso}}$	0.10293	0.08684	0.04010	0.010
H_2O	302	$A_{\text{C=O,iso}}$	0.35400	0.40356	0.28307	0.070
		$A_{\text{C-C,iso}}$	0.10320	0.08695	0.03998	0.024
H_2O^*	302	$A_{\text{C=O,iso}}$	0.25885	0.28970	0.19443	0.070
		$A_{\text{C-C,iso}}$	0.11846	0.10884	0.05290	0.024

3 Computational Inputs and Molecular Geometries

Sample input files:

RSH-PCM

```
$molecule
read                anion.txt
$end

$XC_FUNCTIONAL
X HF        0.20   #short-range exact exchange fraction (alpha)
X wPBE   0.80      #short-range semilocal exchange fraction (beta)
C PBE     1.00
$end

$rem
jobtype                    sp
exchange                gen
correlation               none
lrc_dft                      true
basis                         cc-pVDZ
omega                      193       #Gas-phase tuned result
scf_convergence      8
geom_opt_maxcyc   500
max_scf_cycles        5000
mem_static              4000
mem_total                 48000
magnet                   true        #Required for HFCC calculation
print_general_basis   true
print_orbitals              10
molden_format           true
solvent_method          pcm
$end

$magnet
  hyperfine = true           #Calculates HFCCs
  electric  = true
$end

$pcm
theory              cpcm
method              swig
solver              inversion
HeavyPoints         194
HPoints             194
radii               bondi
vdwscale            1.2
$end

$solvent
Dielectric          7.2
$end
```

SRSH-PCM

```
$molecule
read                anion.txt
$end

$xc_functional
x HF      0.200        #short-range exact exchange fraction (alpha)
x PBE     0.861        #long-range semilocal exchange fraction
x wPBE   -0.061        #beta, where alpha + beta = 1/epsilon
C PBE     1.00
$end

$rem
jobtype                    sp
exchange                gen
correlation               none
lrc_dft                      true
src_dft                      2
basis                         cc-pVDZ
omega                      193         #Gas-phase tuned value
omega2                     193
scf_convergence      8
geom_opt_maxcyc   500
max_scf_cycles        5000
mem_static              4000
hf_lr                      139          #  1/epsilon * 1000
hf_sr                      200          #  alpha * 1000
mem_total                 48000
magnet                   true           #Required for HFCC Calculation
print_general_basis   true
print_orbitals              10
!molden_format           true
solvent_method          pcm
$end

$magnet
  hyperfine = true                      #Calculates HFCCs
  electric  = true
$end

$pcm
theory              cpcm
method              swig
solver              inversion
HeavyPoints         194
HPoints             194
radii               bondi
vdwscale            1.2
$end

$solvent
Dielectric          7.2
$end
```

Figure S2: Sample Q-Chem 6.0 input files for RSH-PCM and SRSH-PCM HFCC calculations.

Coordinates of Optimized Geometries:

Table S19: Optimized coordinates of naphthalene anion.

Atom	X	Y	Z
C	2.4703182066604600	-0.6945625381894300	0.0000000000000019
C	2.4703182070639900	0.6945625368901270	-0.0000000000000050
C	1.2496797234011300	1.4024023994449000	-0.0000000000000019
C	0.0000000001949026	0.7257721398974830	0.0000000000000055
C	-0.0000000002309693	-0.7257721398081760	0.0000000000000070
C	1.2496797226198200	-1.4024024000621200	0.0000000000000095
C	-1.2496797234178300	-1.4024023993559700	-0.0000000000000003
C	-2.4703182070942300	-0.6945625367945200	-0.0000000000000036
C	-2.4703182066916600	0.6945625382707150	0.0000000000000030
C	-1.2496797226369100	1.4024024001517100	0.0000000000000078
H	3.4158546216707800	-1.2450750158829800	0.0000000000000013
H	3.4158546223774700	1.2450750140567900	-0.0000000000000121
H	1.2513588325017300	2.4972360207025800	-0.0000000000000077
H	1.2513588311136900	-2.4972360213216400	0.0000000000000150
H	-1.2513588325276300	-2.4972360206121200	-0.0000000000000056
H	-3.4158546223948300	-1.2450750139834700	-0.0000000000000130
H	-3.4158546216918600	1.2450750159805500	-0.0000000000000003
H	-1.2513588311416900	2.4972360214089000	0.0000000000000069

Table S20: Optimized coordinates of anthracene anion.

Atom	X	Y	Z
C	3.6973707524933100	-0.7017329042451040	-0.0000000000000031
C	3.6973738753940700	0.7017374214335150	0.0000000000000012
C	2.4900536008171000	1.3962347922436400	0.0000000000000008
C	1.2408006454994100	0.7235332509803180	-0.0000000000000010
C	2.4900474099544300	-1.3962249184476800	-0.0000000000000044
C	1.2407974290896200	-0.7235178383424110	-0.0000000000000019
C	-0.0000036264393115	1.4066895948529500	-0.0000000000000014
C	-1.2408108888102000	0.7235387175922340	-0.0000000000000020
C	-0.0000098268058546	-1.4066687220053800	0.0000000000000011
C	-1.2408141058115000	-0.7235123832668070	0.0000000000000016
C	-2.4900608629633600	1.3962457910789100	-0.0000000000000028
C	-3.6973842161430600	0.7017537690473420	-0.0000000000000015
C	-3.6973873389570400	-0.7017165415912570	0.0000000000000015
C	-2.4900670524112900	-1.3962139164483200	0.0000000000000043
H	4.6425344741036000	-1.2508738722963100	-0.0000000000000020
H	4.6425399808421000	1.2508742842181100	0.0000000000000045
H	2.4902958515467300	2.4909261897741700	0.0000000000000054
H	2.4902847991263000	-2.4909163168317900	-0.0000000000000045
H	-0.0000012164950930	2.5017130478217200	0.0000000000000000
H	-0.0000122529807320	-2.5016921747017700	0.0000000000000040
H	-2.4902982814332300	2.4909371852664000	-0.0000000000000046
H	-4.6425479160932800	1.2508947787275700	-0.0000000000000050
H	-4.6425534316900900	-1.2508534275189600	0.0000000000000025
H	-2.4903093400210800	-2.4909053147898600	0.0000000000000073

Table S21: Optimized coordinates of pyrene anion.

Atom	X	Y	Z
C	3.5416196140605900	0.0015351995687536	0.0000000000265780
C	2.8625053585630300	1.2167798653246800	-0.0000000000425957
C	1.4347852675729200	1.2434859744431800	-0.0000000001390109
C	0.7171315254818690	0.0003129725653697	-0.0000000001317687
C	1.4358682240207300	-1.2422392404395000	-0.0000000001386940
C	2.8635648992155000	-1.2142951018126300	-0.0000000000418975
C	0.6910833363305710	2.4496736588287200	-0.0000000001888466
C	-0.6932248556362630	2.4490680114908300	-0.0000000001271020
C	-1.4358761854182000	1.2422363435915700	-0.0000000000386145
C	-0.7171395094436040	-0.0003158859571798	-0.0000000000735219
C	0.6932168515664130	-2.4490709376105200	-0.0000000001888199
C	-0.6910912970530600	-2.4496765853977500	-0.0000000001273949
C	-1.4347932726035500	-1.2434888719026200	-0.0000000000388071
C	-2.8635729198366100	1.2142921887340100	0.0000000000974823
C	-3.5416275964452000	-0.0015380791918141	0.0000000001679641
C	-2.8625133022513300	-1.2167827792854600	0.0000000000972504
H	4.6363333736340600	0.0020137204364041	0.0000000001998012
H	3.4109745687155500	2.1620067588829600	-0.0000000000588505
H	3.4128416860006500	-2.1590536717245100	-0.0000000000573951
H	1.2359273099644700	3.3983705200419400	-0.0000000002517402
H	-1.2388866391540500	3.3972953191991200	-0.0000000001479184
H	1.2388786735887900	-3.3972982256268700	-0.0000000002518149
H	-1.2359353169376800	-3.3983734226569100	-0.0000000001485754
H	-3.4128495967282100	2.1590508236632300	0.0000000001427763
H	-4.6363413559278300	-0.0020167293391180	0.0000000002839647
H	-3.4109826314935200	-2.1620096023147200	0.0000000001423260

Table S22: Optimized coordinates of *p*-benzoquinone in DME using only implicit solvation (PCM).

Atom	X	Y	Z
C	-0.3550737866540490	2.1350821100651200	0.0000000000000000
C	-1.5602635493150800	1.3261122194811900	0.0000000000000000
C	-1.5270540449734700	-0.0429130125675048	0.0000000000000000
C	-0.2845520560371610	-0.7929771066680310	0.0000000000000000
C	0.9206376686998680	0.0159928219602463	0.0000000000000000
C	0.8874281530752050	1.3850180861345100	0.0000000000000000
O	-0.2543651961322560	-2.0542046129609000	0.0000000000000000
O	-0.3852607142669960	3.3963096979225600	0.0000000000000000
H	-2.5138055167233200	1.8639692543843100	0.0000000000000000
H	-2.4536845469028000	-0.6258758193813830	0.0000000000000000
H	1.8741796041294100	-0.5218642742767120	0.0000000000000000
H	1.8140586851006700	1.9679808359065700	0.0000000000000000

Table S23: Optimized coordinates of tetrafluoro-*p*-benzoquinone in DME using only implicit solvation (PCM).

Atom	X	Y	Z
C	0.0512125388528008	2.2399043044447600	0.0000000000000000
C	-1.1256702351494500	1.3961622940068000	0.0000000000000000
C	-1.0712033353140800	0.0335868381310504	0.0000000000000000
C	0.1690241020565680	-0.7137331999272880	0.0000000000000000
C	1.3459067192090200	0.1300087566579850	0.0000000000000000
C	1.2914398185971000	1.4925843100199400	0.0000000000000000
O	0.0010452587971952	3.4900853500895700	0.0000000000000000
O	0.2191910763452430	-1.9639142564179700	0.0000000000000000
F	-2.3196920724870200	2.0159755519103900	0.0000000000000000
F	-2.2120957540918600	-0.6797220963312740	0.0000000000000000
F	2.5399286930641000	-0.4898043052224790	0.0000000000000000
F	2.4323323901203900	2.2058930526385000	0.0000000000000000

Table S24: Optimized coordinates of tetrachloro-*p*-benzoquinone in DME using only implicit solvation (PCM).

Atom	X	Y	Z
C	0.4137556623258190	2.2838662716802300	0.0000000000000000
C	-0.8136761946461650	1.4954025252443900	0.0000000000000000
C	-0.8204733253225590	0.1249762265536780	0.0000000000000000
C	0.3992056409513620	-0.6754567961938520	0.0000000000000000
C	1.6266375648781300	0.1130069188770830	0.0000000000000000
C	1.6334345502256000	1.4834332210865000	0.0000000000000000
O	0.4198617776722160	3.5264766559102200	0.0000000000000000
O	0.3930995181277490	-1.9180671721958000	0.0000000000000000
Cl	-2.2933271501798900	2.4156064352644000	0.0000000000000000
Cl	-2.3091370998707200	-0.7805954002083410	0.0000000000000000
Cl	3.1220984147877900	2.3890048699340200	0.0000000000000000
Cl	3.1062884410506400	-0.8071969559525420	0.0000000000000000

Table S25: Optimized coordinates of *p*-benzoquinone in DMSO using only implicit solvation (PCM).

Atom	X	Y	Z
C	0.6831109848224670	-1.2253338625543500	0.0000000000000000
C	-0.6863009658188230	-1.2241206564978800	0.0000000000000000
C	-1.4636664646221900	0.0012400852999739	0.0000000000000000
C	-0.6841463478222000	1.2252392411379300	0.0000000000000000
C	0.6852656190239590	1.2240259968679100	0.0000000000000000
C	1.4626310324802000	-0.0013347121540772	0.0000000000000000
O	2.7249251579096600	-0.0024385044706961	0.0000000000000000
O	-2.7259604638281600	0.0023438439117086	0.0000000000000000
H	1.2419545601099900	-2.1666333051229500	0.0000000000000000
H	-1.2467917851973300	-2.1644395098826300	0.0000000000000000
H	-1.2429899841723800	2.1665386531678600	0.0000000000000000
H	1.2457563191590200	2.1643449163978800	0.0000000000000000

Table S26: Optimized coordinates of *p*-benzoquinone in H_2O using only implicit solvation (PCM).

Atom	X	Y	Z
C	0.6831088683538410	-1.2253699085997200	0.0000000000000000
C	-0.6862989572322170	-1.2241567093128500	0.0000000000000000
C	-1.4635667785809300	0.0012400010197507	0.0000000000000000
C	-0.6841442312817340	1.2252752872395200	0.0000000000000000
C	0.6852636104734810	1.2240620496450100	0.0000000000000000
C	1.4625313463481000	-0.0013346278967690	0.0000000000000000
O	2.7248798239091000	-0.0024384494634683	0.0000000000000000
O	-2.7259151295537700	0.0023437889753592	0.0000000000000000
H	1.2418774277511900	-2.1667096831227200	0.0000000000000000
H	-1.2467147918274900	-2.1645160470871600	0.0000000000000000
H	-1.2429128520184900	2.1666150310689500	0.0000000000000000
H	1.2456793257031500	2.1644214536347500	0.0000000000000000

Table S27: Optimized coordinates of *p*-benzoquinone in H_2O using implicit solvation (PCM) and two explicit H_2O molecules.

Atom	X	Y	Z
C	-2.0462197072569800	1.5693711877395300	-0.1797579860020890
C	-1.5898059933978600	0.3082239894689120	0.0906974698957786
C	-1.1564819454657900	2.7061410784268800	-0.2692512441319140
C	0.2482271971878320	2.4329232385228600	-0.0502265777337108
C	0.7046155879952980	1.1715968620589400	0.2202761294234180
C	-0.1847404215453540	0.0341097658041626	0.3061419488125180
O	-1.5913055142818700	3.8712432011658700	-0.5281137815177260
O	0.2503761052842120	-1.1325196157678100	0.5579998252265350
H	-3.1114802637414000	1.7584628130700100	-0.3401337422172410
H	-2.2745823523687600	-0.5410229744906390	0.1564620717526210
H	0.9322130969510010	3.2831378357703000	-0.1146759492371800
H	1.7698546629572700	0.9819581923033600	0.3809304407082320
O	0.4400738947336130	5.7056427115981800	-0.4319943338746190
H	-0.3170420178769870	5.0810035442224200	-0.5256362867443000
H	0.5157528755928450	5.7797288015485400	0.5274373621995670
O	-1.7768967102184700	-2.9693439577258900	0.3827555007249870
H	-1.9122144122282700	-2.9444610096946600	-0.5726039958287610
H	-1.0183617828504000	-2.3498775640529300	0.4961761495052340
